# Differential effects of bariatric surgery and lifestyle interventions on plasma levels of Lp(a) and fatty acids

**DOI:** 10.1186/s12944-022-01756-1

**Published:** 2022-12-28

**Authors:** Kirsten A. Berk, Heidi Borgeraas, Ingunn Narverud, Monique T. Mulder, Linn K. L. Øyri, Adrie J. M. Verhoeven, Milada Cvancarova Småstuen, Martin P. Bogsrud, Torbjørn Omland, Jens Kristoffer Hertel, Espen Gjevestad, Njord Nordstrand, Kirsten B. Holven, Jøran Hjelmesæth

**Affiliations:** 1 Department of Internal Medicine, Division of Pharmacology and Vascular Medicine, Erasmus Medical Center, Rotterdam, the Netherlands; 2 Department of Internal Medicine, Division of Dietetics, Erasmus Medical Center, Rotterdam, The Netherlands, Erasmus University MC, Rotterdam, The Netherlands; 3 Morbid Obesity Center, Vestfold Hospital Trust, Tønsberg, Norway; 4 Norwegian National Advisory Unit On Familial Hypercholesterolemia, Department of Endocrinology, Morbid Obesity and Preventive Medicine, Oslo University Hospital, Oslo, Norway; 5 Department of Nutrition, Institute of Basic Medical Sciences, University of Oslo, Oslo, Norway; 6 Department of Nutrition and Management, Oslo Metropolitan University, Oslo, Norway; 7 Unit for Cardiac and Cardiovascular Genetics, Oslo University Hospital, Oslo, Norway; 8 Department of Cardiology, Akershus University Hospital, Lørenskog, Norway; 9 K.G. Jebsen Center of Cardiac Biomarkers, Institute of Clinical Medicine, University of Oslo, Oslo, Norway; 10 Division of Physical Medicine and Rehabilitation, Vestfold Hospital Trust, Stavern, Norway; 11 Norwegian Police University College, Stavern, Norway; 12 Department of Endocrinology, Morbid Obesity and Preventive Medicine, Institute of Clinical Medicine, University of Oslo, Oslo, Norway

**Keywords:** Lp(a), Fatty acids, Obesity, Bariatric surgery, Lifestyle intervention

## Abstract

**Background:**

Limited evidence suggests that surgical and non-surgical obesity treatment differentially influence plasma Lipoprotein (a) [Lp(a)] levels. Further, a novel association between plasma arachidonic acid and Lp(a) has recently been shown, suggesting that fatty acids are a possible target to influence Lp(a). Here, the effects of bariatric surgery and lifestyle interventions on plasma levels of Lp(a) were compared, and it was examined whether the effects were mediated by changes in plasma fatty acid (FA) levels.

**Methods:**

The study includes two independent trials of patients with overweight or obesity. Trial 1: Two-armed intervention study including 82 patients who underwent a 7-week low energy diet (LED), followed by Roux-en-Y gastric bypass and 52-week follow-up (surgery-group), and 77 patients who underwent a 59-week energy restricted diet- and exercise-program (lifestyle-group). Trial 2: A clinical study including 134 patients who underwent a 20-week very-LED/LED (lifestyle-cohort).

**Results:**

In the surgery-group, Lp(a) levels [median (interquartile range)] tended to increase in the pre-surgical LED-phase [17(7–68)-21(7–81)nmol/L, *P* = 0.05], but decreased by 48% after surgery [21(7–81)—11(7–56)nmol/L, *P* < 0.001]. In the lifestyle-group and lifestyle-cohort, Lp(a) increased by 36%[14(7–77)—19(7–94)nmol/L, *P* < 0.001] and 14%[50(14–160)—57(19–208)nmol/L, *P* < 0.001], respectively. Changes in Lp(a) were independent of weight loss. Plasma levels of total saturated FAs remained unchanged after surgery, but decreased after lifestyle interventions. Arachidonic acid and total n-3 FAs decreased after surgery, but increased after lifestyle interventions. Plasma FAs did not mediate the effects on Lp(a).

**Conclusion:**

Bariatric surgery reduced, whereas lifestyle interventions increased plasma Lp(a), independent of weight loss. The interventions differentially influenced changes in plasma FAs, but these changes did not mediate changes in Lp(a).

**Trial registration:**

Trial 1: Clinicaltrials.gov NCT00626964.

Trial 2: Netherlands Trial Register NL2140 (NTR2264).

**Graphical abstract:**

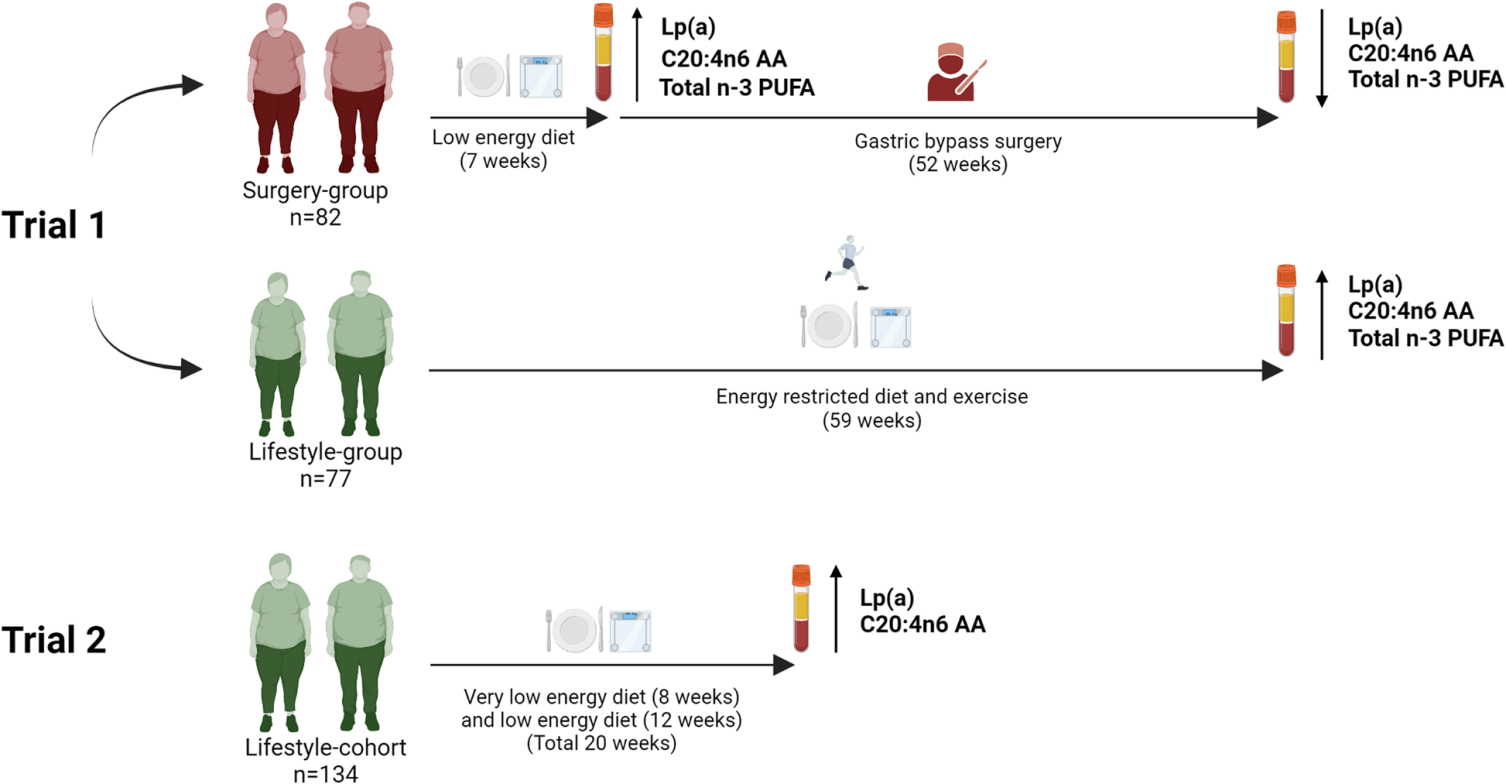

**Supplementary Information:**

The online version contains supplementary material available at 10.1186/s12944-022-01756-1.

## Introduction

Lipoprotein (a) [(Lp(a)] is a low density lipoprotein (LDL)-like particle with an apolipoprotein (a) [apo(a)] attached to the ApoB_100_, and is considered an independent risk factor for cardiovascular disease [1–4]. Plasma Lp(a) levels can be manipulated, and studies indicate that as much as 25% of the variance in Lp(a) levels is ascribed to lifestyle factors [5]. However, the mechanisms by which Lp(a) levels are regulated are not well understood.

Lp(a) levels are influenced by caloric restriction and bariatric surgery, but do not appear to be regulated by weight loss per se [6]. It has previously been shown that weight loss after energy-restricted dieting was associated with an increase in plasma Lp(a) levels in adults with or without type 2 diabetes (T2D), while plasma Lp(a) levels showed a strong tendency to decrease in patients without T2D who underwent bariatric surgery [7]. A recent meta analysis showed that bariatric surgery significantly decreased circulating Lp(a) levels, and that the decrease in Lp(a) was not associated with change in body mass index (BMI) [8].

Lp(a) levels may also be regulated, to some extent, by changes in plasma fatty acids (FAs). A positive, novel association between plasma levels of the n-6 FA arachidonic acid (AA) and Lp(a) in patients with familial hypercholesterolemia has recently been shown [9]. Other studies have shown that an increased intake of total- and saturated fat is accompanied by a decrease in Lp(a) levels [10–13], and that supplementation of conjugated LA lead to increased Lp(a) levels [14]. The composition of the plasma FA pool may be altered both by caloric restriction [15] and bariatric surgery [16–18], where possible contributing factors are the reduced dietary intake, changes in the dietary composition, malabsorption of lipids [19] and release of FAs from the body fat deposits during weight loss. Whether changes in plasma Lp(a) levels following caloric restriction or bariatric surgery are mediated by changes in plasma FA levels is not known. It is important to identify opportunities to reduce adverse changes to Lp(a) during weight loss dieting, through intervening on plasma FAs.

In this study, the effects of Roux-en-Y gastric bypass surgery (RYGB) and an intensive lifestyle intervention, including caloric restriction and exercise, on plasma Lp(a) and FA levels in patients with obesity were compared, and it was also examined whether possible effects on plasma Lp(a) levels were mediated by changes in plasma FA levels. The effects of a lifestyle intervention on plasma Lp(a) and FA levels in an independent cohort of patients with T2D and overweight or obesity were also examined.

## Methods

### Study subjects and design

This study includes two independent trials.

*Trial 1* is a two-armed non-randomized study which compared the 1-year effects of RYGB (surgery-group) with intensive lifestyle intervention (lifestyle-group) (Clinicaltrials.gov NCT00626964), conducted at the Morbid Obesity Centre, Vestfold Hospital Trust, Tønsberg, Norway between February 2008 and February 2011. Inclusion criteria were BMI ≥ 40 kg/m^2^, or ≥ 35 kg/m^2^ and at least one obesity related comorbidity. The primary outcome (arterial stiffness) and data on weight-loss and changes in metabolic biomarkers have previously been published [20, 21].

*Trial 2* includes individuals who participated in the Prevention Of Weight Regain (POWER) cohort study [Netherlands Trial Register NL2140 (NTR2264)] (lifestyle-cohort) [22]. Participants were recruited at the outpatient diabetes clinic of the Erasmus University Medical Centre, Rotterdam, The Netherlands, between March 2010 and April 2015. The inclusion criteria were BMI > 27 kg/m^2^ and T2D. The primary outcome (Lp(a) levels) and data on weight-loss and changes in metabolic biomarkers have previously been published [7].

Trial 1 was approved by the The Regional Committees for Medical and Health Research Ethics in Norway (code: S-05175) and trial 2 was approved by the Medical Ethics Committee of the Erasmus Medical Center (reference numbers MEC-2009–143, MEC-2014–090 and MEC 2016–604). Both trials were conducted according to the principles in the Declaration of Helsinki, and written informed consent was provided by all the participants.

### Interventions

*Trial 1:* The participants in the surgery-group followed a low energy diet (LED) (< 900 kcal per day) for 7 weeks prior to surgery (pre-surgery phase), and were followed for 52 weeks after surgery (post-surgery phase) where they received standard follow-up care at the Morbid Obesity Centre—a total follow-up of 59 weeks.

The participants in the lifestyle-group underwent a dietary and physical activity intervention which lasted for a total of 59 weeks [20, 21]. They received nutritional counseling according to Norwegian nutritional guidelines and every participant’s energy intake was reduced by 1000 kcal/day, they also underwent 90 min supervised training sessions, including weight bearing and aerobic exercise, 3 days/week during the first 12 weeks. Thereafter, the participants received monthly follow-ups, and were advised to maintain physical activity for 60–90 min per day throughout the study period (59 weeks).

*Trial 2:* The participants underwent a dietary intervention which lasted for a total of 20 weeks. During the first 8 weeks, the participants followed a very LED of approximately 750 kcal per day, which consisted of two meal replacements (Glucerna SR, Abbott Nutrition, Lake Forest, Illinois, USA), plus a small dinner, providing a total of 67 g carbohydrates, 11.5 g of fibre, 54 g protein and 32 g fat (of which 16 g monounsaturated FAs) daily and micronutrients according to Recommended Dietary Allowance (RDA) recommendations. Thereafter, energy intake was slowly increased over 12 weeks up to approximately 1300 kcal per day. In addition, 30–60 min of daily exercise was encouraged during the entire intervention.

### Outcomes

The main outcomes were plasma levels of Lp(a) and FAs. Plasma levels were measured at baseline (trial 1 and 2), 7 and 59 weeks (trial 1), and at 20 weeks (trial 2).

### Laboratory analyses

In trial 1, plasma Lp(a) concentrations were measured using a particle-enhanced immunoturbidimetric method, by Roche Diagnostics at an accredited medical laboratory, Oslo University Hospital, Rikshospitalet, Oslo, Norway (NS-EN ISO 15189:2007). The samples were stored for 6–9 years at -80 °C, and had not been thawed prior to the Lp(a) analysis. In trial 2 Lp(a) concentrations were measured using the Diagnostic System #171,399,910,930 (DiaSys Diagnostic System, GmbH, Holzheim, Germany). The samples were stored for 5–10 years at -80 °C before analysis, and had not been thawed prior to the Lp(a) analysis. Plasma Lp(a) levels were subsequently re-measured in a sub-group of participants from trial 2 using Roche Diagnostics to evaluate method agreement. Measurements of fasting serum blood glucose and lipoprotein profiles have been described previously [21, 22].

Plasma free FA profiles were determined by Gas Chromatography-Flame Ionization Detector analysis at the commercial laboratory Vitas Analytical Services. The serum samples were thawed and aliquoted to dried blood spot (DBS) paper (Whatman 903 paper) until GC-analysis. One 4.7 mm punch of human plasma DBS paper were methylated with sodium methoxide in methanol. After methylation, FA methyl esters (FAME) were extracted with hexane. After thorough mixing and centrifugation, 3 µl of the hexane phase was injected into the GC-FID. GC-FID was performed with an Agilent 7890A Gas Chromatograph System (Agilent Technologies, Palo Alto, CA, USA). Separations was performed on a SP-2380 (30 m × 0.25 mm i.d. × 0.25 µm film thickness) column from Supelco. The results are shown as percentages of total FAs. In trail 1, the samples were stored for 7–10 years at -80 °C, and were frozen twice before FA analysis. In trial 2, the samples were stored for 5–10 years at -80 °C and had not been thawed prior to FA analysis.

### Statistical analyses

Data are presented as means [standard deviation (SD)] or medians [interquartile range (IQR)] for continuous data, and as counts (%) for categorical data. McNemar’s test, paired T-test or Wilcoxon Signed Rank test were used when investigating within-group changes.

Statistical between-group comparisons were made between the lifestyle-group of trail 1 (baseline-59 weeks) and the surgery-group of trial 1 (week 7–59). Between-group differences in changes from baseline (lifestyle-group) and from week 7 (surgery-group) to end of intervention were estimated using a robust linear regression approach which is a non-parametric iterative method using weights from absolute residuals. The results are expressed as means (95% CI), and STATA version 15.0 was used to perform the analyses. Mediation analyses were performed using the PROCESS macro (version 3.3) for SPSS written by A F Hayes [23], with group (lifestyle vs. surgery) as the independent variable, change in Lp(a) level (7 weeks to 59 weeks for the surgery-group and baseline to 59 weeks for the lifestyle-group) as the dependent variable and change in FA level (7 weeks to 59 weeks for the surgery-group and baseline to 59 weeks for the lifestyle-group) as the mediator variable. Hayes uses the three steps as originally suggested by Byron and Kenny. In step 1 the independent variable is regressed on the mediator. In step 2 the independent variable is regressed on the dependent variable. In step 3 the final model with the independent variable and the moderator as covariates is fitted and the proportion of the association between the independent and dependent variable which is explained by the mediator can be calculated. *P*-values in the mediation analyses were calculated using the Sobel test. *P*-values < 0.05 were considered statistically significant. The analyses were considered exploratory, thus no corrections for multiple testing were performed.

## Results

### Characteristics of the participants

**Fig. 1:**
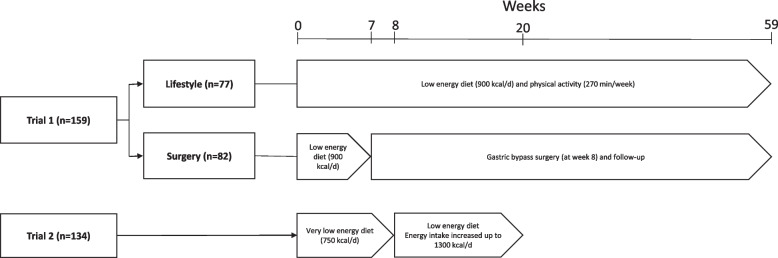
Flowchart of trial 1 and 2 depicting included patients, interventions and length of follow-up

In trial 1, 82 of the 98 patients in the surgery-group, and 77 of the 102 patients in the lifestyle-group completed the 59-week follow-up, leaving 159 patients to be included in the present analysis (Fig. 1). Trial 2 (lifestyle-cohort) included 161 participants, whereof the 134 participants who had measured plasma Lp(a) and FAs before and after the intervention were included in the current analysis.

The baseline characteristics of participants in both trials are presented in Table 1. In trial 1, more than 60% (*n* = 102) of the participants in both arms were female, and 97% (*n* = 155) were White. The participants in the surgery-group were younger (41 years vs. 47 years, *P* = 0.011), had a higher BMI (46 kg/m^2^ vs. 42 kg/m^2^, *P* < 0.001), and were less often diagnosed with cardiovascular disease (2% vs. 16%, *P* = 0.004), compared with participants in the lifestyle-group. A total of 26% (*n* = 41) had T2D, and 19% (*n* = 30) were prescribed a statin, with no difference between the groups. In trial 2, 60% (*n* = 80) of the participants were women and 45% (*n* = 73) were White, the median age was 55 years and the mean BMI 35 kg/m^2^. All participants were diagnosed with T2D, 49% (*n* = 65) were on insulin treatment, 16% (*n* = 22) were diagnosed with cardiovascular disease and 58% (*n* = 77) received statin treatment.

**Table 1:** Characteristics of the participants at baseline and after interventions

	**Trial 1**								**Trial 2**		
	**Surgery-group *n* = 82**					**Lifestyle-group *n* = 77**			**Lifestyle-cohort *n* = 134**		
	**Baseline**	**7 weeks**	P^*^	**59 weeks**	***P***^**†**^	**Baseline**	**59 weeks**	***P***^**‡**^	**Baseline**	**20 weeks**	***P***^**‡**^
Age, years	41 (36–49)					47 (37–55)			55 (26–74)		
Sex, female, n (%)	54 (65.9)					48 (62.3)			80 (59.7)		
Ethnicity, white, n (%)	80 (97.6)					75 (97.4)			73 (45)		
Cardiovascular disease, n (%)	2 (2)					12 (16)			22 (16.4)		
Type 2 diabetes mellitus, n (%)	24 (29.3)					17 (22.1)			134 (100)		
Statins, n (%)	12 (14.6)	11 (13.4)	1.00	9 (11.0)	0.63	18 (23.4)	19 (24.7)	1.00	77 (58.0)	75 (56.0)	1.00
Insulin, n (%)	1 (1.2)	1 (1.2)	1.00	0 (0)	1.00	4 (5.2)	3 (3.9)	1.00	65 (48.5)	47 (35.1)	0.125
BMI, kg/m2	45.6 (5.3)	42.6 (5.0)	**< 0.001**	31.2 (4.7)	**< 0.001**	42.0 (4.9)	38.0 (5.9)	**< 0.001**	35.4 (32.4–38.6)	32.3 (29.4–35.8)	**< 0.001**
Weight, kg	137.1 (21.9)	127.9 (20.1)	**< 0.001**	93.8 (17.7)	**< 0.001**	124.2 (20.0)	112.2 (21.3)	**< 0.001**	101.5 (89.4–115.8)	92.1 (80.8–105.2)	**< 0.001**
Total cholesterol, mmol/L	4.9 (1.0)	4.6 (1.0)	**< 0.001**	4.3 (0.9)	**< 0.001**	5.1 (1.0)	5.0 (1.1)	0.57	4.4 (3.6–5.1)	4.1 (3.5–4.9)	**0.001**
HDL cholesterol, mmol/L	1.1 (0.3)	1.0 (0.2)	**< 0.001**	1.5 (0.4)	**< 0.001**	1.2 (0.3)	1.3 (0.4)	**< 0.001**	1.2 (1.0–1.4)	1.2 (1.0–1.4)	**0.019**
LDL cholesterol, mmol/L	3.1 (0.9)	2.9 (0.9)	**0.014**	2.4 (0.7)	**< 0.001**	3.1 (0.9)	3.1 (1.0)	0.98	2.5 (2.1–3.2)	2.4 (1.7–2.9)	**0.003**
Triglycerides, mmol/L	1.5 (1.0–2.0)	1.2 (0.9–1.7)	**< 0.001**	0.9 (0.7–1.1)	**< 0.001**	1.4 (1.1–2.0)	1.2 (0.8–1.5)	**< 0.001**	1.6 (1.1–2.4)	1.4 (1.0–1.9)	**< 0.001**
Lp(a), nmol/L	16.5 (7.0–68.0)	21.0 (7.0–81.3)	0.05	10.5 (7.0–55.8)	**< 0.001**	14 (7–77)	19 (7–94)	**< 0.001**	50 (14–160)	57 (19–208)	**< 0.001**
Glucose, mmol/L	5.4 (5.1–6.3)	5.1 (4.7–5.8)	**0.001**	4.7 (4.5–5.0)	**< 0.001**	5.3 (4.9–6.3)	5.1 (4.6–5.8)	**0.036**	8.5 (6.9–10.5)	7.2 (6.1–9.0)	**< 0.001**
C-reactive protein, mg/dL	8.1 (3.7–12.0)	5.2 (3.0–9.2)	**< 0.001**	0.6 (0.7–1.8)	**< 0.001**	4.8 (2.5–8.7)	2.8 (1.4–5.8)	**< 0.001**	4.5 (1.6–15.0)	3.4 (1.1–8.8)	**0.009**

*Abbreviations*: *BMI* body mass index, *HDL* high density lipoprotein, *LDL* low density lipoprotein. Data are given as mean (standard deviation) or median (interquartile range). McNemar’s test, paired T test or Wilcoxon signed Rank test comparing

^*^baseline and 7 weeks

^†^7 weeks and end of intervention

^‡^baseline and end of intervention

### Weight loss and changes in metabolic biomarkers

In trial 1, the initial 7-week LED in the surgery-group led to a mean (95% CI) total body weight loss (TBWL) of 7 (6–7)%, followed by an additional 27 (25–28)% TBWL after surgery (week 7–59) (Table 1). The lifestyle-group had a TBWL of 10 (8–12)% at 59-week follow-up. The participants in trial 2 (lifestyle-cohort) had a TBWL of 9 (8–10)% at 20 weeks follow-up.

The serum levels of triglycerides, fasting glucose and C-reactive protein decreased significantly over time in both groups in trial 1 and also in trial 2 (Table 1). Serum total cholesterol and LDL cholesterol remained unchanged in the lifestyle-group in trial 1, but decreased significantly after surgery in trial 1 and also in trial 2. High density lipoprotein (HDL) cholesterol levels increased in both groups in trial 1 and also in trial 2.

### Lipoprotein (a)

**Fig. 2:**
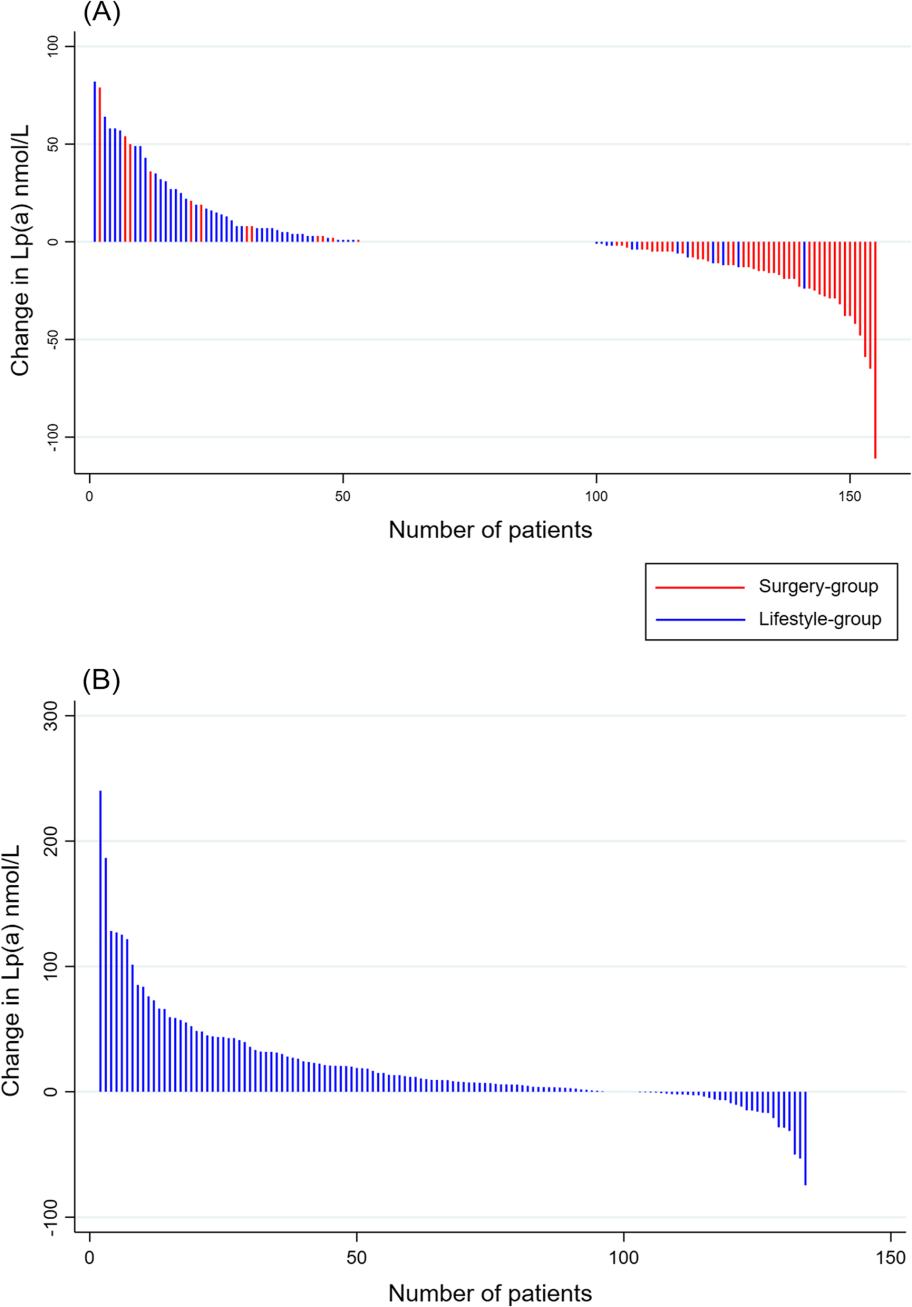
Waterfall plot depicting changes in Lp(a) levels from 7 to 59 weeks for each individual in the surgery-group and from baseline to 59 weeks for each individual in lifestyle-group of trial 1 (panel **A**), and from baseline to 20 weeks for each individual in trial 2 (panel **B**)

In the surgery-group, the median (IQR) concentration of Lp(a) tended to increase during the 7-week pre-surgery LED-phase [from 17 (7–68) to 21 (7–81) nmol/L, *P* = 0.05], but were decreased by 48% after surgery [from 21 (7–81) to 11 (7–56) nmol/L, P < 0.001] at week 59 (Table 1). There was also a significant 35% decrease in plasma Lp(a) levels when comparing baseline values to values at 59 weeks [from 17 (7–68) to 11 (7–56) nmol/L, *P* = 0.004] in the surgery-group. Median plasma levels of Lp(a) increased by 36% [from 14 (7–77) to 19 (7–94) nmol/L, *P* < 0.001] during 59-week follow-up in the lifestyle-group (Trial 1). There was a significant difference in change [mean (95% CI)] in Lp(a) levels when comparing the surgery-group (week 7–59) with the lifestyle-group (baseline-59 weeks) [-8.0 (-11.1, -4.8) nmol/L, *P* < 0.001], and adjusting for changes in body weight, sex and age did not significantly influence the results (data not shown). Changes in Lp(a) levels from week 7 to week 59 for each individual participant in the surgery-group and from baseline to 59 weeks for the participants in the lifestyle-group, are presented in Fig. 2A. The figure shows that the majority of participants in the surgery group experienced a reduction in plasma Lp(a) levels, while the majority of the participants in the lifestyle-group experienced increased plasma levels of Lp(a) during follow-up. In trial 2 (lifestyle-cohort), median plasma levels of Lp(a) increased by 14% [from 50 (14–160) to 57 (19–208) nmol/L, *P* < 0.001] during 20-week follow-up. Changes in Lp(a) levels from baseline to 20 weeks for the participants in trial 2 are presented in Fig. 2B. There was no significant association between change in Lp(a) levels and change in body weight in trial 2 (data not shown).

### Fatty acids

#### Saturated fatty acids

In trial 1, plasma levels of total saturated FAs did not change after surgery (week 7–59), but decreased slightly after the 59-week lifestyle-intervention (Table 2). Plasma levels of myristic acid (C14:0), pentadecylic acid (C15:0) and stearic acid (C18:0) increased after surgery, but decreased (myristic acid) or remained unchanged (pentadecylic acid and stearic acid) in the lifestyle-group. Palmitic acid (C16:0) levels decreased in both groups. Plasma levels of all the individual saturated FAs changed significantly more after surgery than after the lifestyle intervention (Table 3).

**Table 2:** Plasma levels of saturated-, monounsaturated- and polyunsaturated fatty acids in trial 1 and trial 2

	**Trial 1**								**Trial 2**		
	**Surgery-group *n* = 82**					**Lifestyle-group *n* = 77**			**Lifestyle-cohort *n* = 134**		
	**Baseline**	**7 weeks**	P^*^	**59 weeks**	***P***^†^	**Baseline**	**59 weeks**	***P***^‡^	**Baseline**	**20 weeks**	***P***^‡^
**SFAs, % of total fatty acids**											
Total SFA	30.4 (29.1–31.9)	29.6 (28.4–30.5)	**< 0.001**	29.4 (28.5–30.1)	0.36	30.5 (29.3–32.0)	29.8 (29.0–30.9)	**0.001**	32.5 (30.6–34.0)	31.0 (29.6–32.8)	**< 0.001**
Myristic acid, 14:0	0.90 (0.70–1.20)	0.50 (0.40–0.70)	**< 0.001**	0.70 (0.55–0.90)	**< 0.001**	1.00 (0.80–1.20)	0.80 (0.70–1.10)	**0.001**	1.09 (0.81–1.39)	0.88 (0.70–1.19)	**< 0.001**
Pentadecylic acid, 15:0	0.20 (0.20–0.30)	0.20 (0.20–0.30)	0.32	0.30 (0.20–0.30)	**< 0.001**	0.20 (0.20–0.30)	0.20 (0.20–0.30)	0.45	0.19 (0.15–0.22)	0.18 (0.16–0.22)	0.52
Palmitic acid, 16:0	22.3 (21.3–24.0)	22.7 (21.7–23.5)	0.98	21.6 (20.8–22.3)	**< 0.001**	22.2 (21.3–23.8)	21.9 (21.1–23.3)	**0.01**	22.8 (21.2–24.2)	22.0 (20.5–23.1)	**< 0.001**
Stearic acid, C18:0	6.90 (6.30–7.30)	6.00 (5.60–6.40)	**< 0.001**	6.80 (6.35–7.15)	**< 0.001**	6.80 (6.40–7.10)	6.80 (6.20–7.20)	0.76	6.62 (6.09–7.06)	6.48 (6.03–6.95)	0.26
**MUFAs, % of total fatty acids**											
Total MUFA	26.3 (24.0–28.4)	26.1 (23.1–28.5)	0.66	25.6 (24.2–28.3)	0.39	25.2 (22.8–27.9)	24.2 (22.9–27.1)	0.21	24.8 (20.8–28.8)	24.3 (21.1–28.5)	0.15
Palmitoleic acid, 16:1 n-7	2.45 (2.10–3.00)	2.10 (1.70–2.50)	**< 0.001**	1.90 (1.45–2.10)	**< 0.001**	2.30 (1.90–2.70)	2.10 (1.70–2.40)	**< 0.001**	2.03 (1.52–2.75)	1.60 (1.25–2.18)	**< 0.001**
Oleic acid, 18:1 n-9	21.9 (20.2–23.3)	21.9 (19.5–24.1)	0.69	21.8 (20.7–23.8)	0.52	21.0 (19.2–23.5)	20.5 (19.4–23.4)	0.40	21.1 (17.7–24.3)	21.0 (18.1–24.3)	0.91
Vaccenic acid, 18:1 n-7 cis	1.60 (1.50–1.80)	1.90 (1.70–2.00)	**< 0.001**	1.80 (1.60–1.90)	**< 0.001**	1.60 (1.40–1.70)	1.60 (1.50–1.80)	0.12	1.41 (1.22–1.65)	1.41 (1.27–1.64)	0.42
Eicosenoic acid, 20:1 n-9	0.10 (0.10–0.20)	0.10 (0.10–0.20)	0.67	0.20 (0.10–0.20)	**< 0.001**	0.10 (0.10–0.20)	0.20 (0.10–0.20)	**0.002**	0.14 (0.11–0.18)	0.15 (0.12–0.18)	0.08
**n-6 PUFAs, % of total fatty acids**											
Total n-6 PUFAs	34.5 (30.8–38.1)	35.4 (32.9–38.2)	**0.009**	35.5 (33.0–37.8)	0.34	35.4 (32.2–39.4)	36.2 (33.8–39.1)	0.21	33.5 (29.4–37.1)	35.0 (30.7–38.5)	**< 0.001**
LA, C18:2 n-6	26.7 (23.0–29.8)	26.5 (24.2–28.9)	0.53	27.1 (24.3–28.8)	**0.038**	27.1 (24.9–30.4)	27.6 (25.2–29.9)	0.98	23.9 (20.6–27.3)	25.1 (21.4–28.4)	**0.007**
GLA, C18:3 n-6	0.40 (0.30–0.50)	0.20 (0.20–0.30)	**< 0.001**	0.30 (0.20–0.40)	**0.025**	0.40 (0.40–0.50)	0.40 (0.30–0.50)	**0.004**	0.43 (0.35–0.58)	0.42 (0.33–0.55)	0.11
EDA, C20:2 n-6	0.20 (0.20–0.20	0.20 (0.20–0.20)	0.17	0.20 (0.20–0.30)	**< 0.001**	0.20 (0.20–0.30)	0.20 (0.20–0.20)	0.84	0.25 (0.23–0.28)	0.26 (0.23–0.30)	**0.025**
DGLA, C20:3 n-6	1.60 (1.40–1.90)	1.20 (1.10–1.50)	**< 0.001**	1.50 (1.30–1.80)	**< 0.001**	1.70 (1.40–1.90)	1.60 (1.40–1.90)	0.97	1.45 (1.17–1.68)	1.39 (1.11–1.60)	**0.042**
AA, C20:4 n-6	5.70 (5.00–6.80)	7.30 (6.10–8.30)	**< 0.001**	6.70 (5.90–7.60)	**< 0.001**	5.70 (4.90–6.80)	6.50 (5.50–7.60)	**< 0.001**	6.72 (5.50–7.79)	7.54 (6.06–8.54)	**< 0.001**
**n-3 PUFAs, % of total fatty acids**											
Total n-3 PUFAs	3.80 (3.20–4.63)	4.90 (3.90–6.30)	**< 0.001**	4.20 (3.80–4.90)	**< 0.01**	4.50 (3.55–5.80)	4.90 (3.90–6.45)	**0.001**	3.67 (3.24–4.37)	3.85 (3.34–4.30)	0.46
ALA, C18:3 n-3	0.60 (0.50–0.70)	0.50 (0.40–0.60)	**< 0.001**	0.50 (0.40–0.60)	0.14	0.60 (0.50–0.80)	0.60 (0.50–0.70)	**0.001**	0.55 (0.42–0.75)	0.55 (0.43–0.73)	0.56
EPA, C20:5 n-3	0.80 (0.50–1.20)	1.00 (0.60–1.70)	**< 0.001**	0.80 (0.60–1.20)	**0.021**	0.90 (0.70–1.60)	1.10 (0.70–1.70)	**0.037**	0.72 (0.51–0.98)	0.68 (0.47–0.99)	0.16
DPA, C22:5 n-3	0.50 (0.50–0.60))	0.60 (0.50–0.70)	**< 0.001**	0.70 (0.60–0.80)	**< 0.001**	0.60 (0.50–0.70)	0.60 (0.60–0.70)	**0.004**	0.50 (0.43–0.58)	0.50 (0.42–0.57)	0.28
DHA, C22:6 n-3	1.90 (1.50–2.60)	2.80 (2.20–3.50)	**< 0.001**	2.30 (1.90–2.80)	**< 0.001**	2.40 (1.70–2.80)	2.60 (2.00–3.20)	**< 0.001**	1.86 (1.56–2.26)	1.99 (1.58–2.32)	0.11

*Abbreviations*: *AA* arachidonic acid, *ALA* alpha linolenic acid, *DGLA* dihomo-gamma-linolenic acid, *DHA* docosahexaenoic acid, *DPA* docosapentaenoic, *EDA* eicosadienoic acid, *EPA* eicosapentaenoic acid, *GLA* gamma-linolenic acid, *LA* linoleic acid, *MUFA* monounsaturated fatty acids, *SFA* saturated fatty acids, *PUFA* polyunsaturated fatty acids. Data are given as mean (standard deviation) or median (interquartile range). Paired T test or Wilcoxon signed Rank test comparing

^*^baseline and 7 weeks

^†^7 weeks and end of intervention

^‡^baseline and end of intervention

**Table 3:** Differences in changes of plasma levels of fatty acids between the surgery-group (7–59 weeks) and the lifestyle-group (baseline-59 weeks) of trial 1^a^

	**Mean difference (95% CI)**	***P***^†^
**SFAs, % of total fatty acids**		
Total SFA	0.44 (-0.01, 0.89)	0.06
Myristic acid, 14:0	0.31 (0.21, 0.42)	**< 0.001**
Pentadecylic acid, 15:0	0.04 (0.02, 0.06)	**< 0.001**
Palmitic acid, 16:0	-0.62 (-1.07, -0.18)	**0.006**
Stearic acid, C18:0	0.75 (0.51, 0.99)	**< 0.001**
**MUFAs, % of total fatty acids**		
Total MUFA	0.14 (-0.73, 1.02)	0.75
Palmitoleic acid, 16:1 n-7	-0.05 (-0.23, 0.12)	0.53
Oleic acid, 18:1 n-9	0.39 (-0.34, 1.13)	0.29
Vaccenic acid, 18:1 n-7 cis	-0.18 (-0.26, -0.10)	**< 0.001**
Eicosenoic acid, 20:1 n-9	0.00 (0.00, 0.00)	1.00
**n-6 PUFAs, % of total fatty acids**		
Total n-6 PUFAs	-0.28 (-1.48, 0.92)	0.65
LA, C18:2 n-6	0.76 (-0.33, 1.85)	0.17
GLA, C18:3 n-6	0.09 (0.04, 0.13)	**< 0.001**
EDA, C20:2 n-6	0.05 (0.03, 0.06)	**< 0.001**
DGLA, C20:3 n-6	0.27 (0.15, 0.38)	**< 0.001**
AA, C20:4 n-6	-1.15 (-1.55, -0.76)	**< 0.001**
**n-3 PUFAs, % of total fatty acids**		
Total n-3 PUFAs	-0.98 (-1.44, -0.52)	**< 0.001**
ALA, C18:3 n-3	0.10 (0.05, 0.15)	**< 0.001**
EPA, C20:5 n-3	-0.32 (-0.54, -0.10)	**0.005**
DPA, C22:5 n-3	0.04 (0.00, 0.08)	**0.042**
DHA, C22:6 n-3	-0.70 (-0.91, -0.48)	**< 0.001**

*Abbreviations*: *AA* arachidonic acid, *ALA* alpha linolenic acid, *DGLA* dihomo-gamma-linolenic acid, *DHA* docosahexaenoic acid, *DPA* docosapentaenoic, *EDA* eicosadienoic acid, *EPA* eicosapentaenoic acid, *GLA* gamma-linolenic acid, *LA* linoleic acid, *MUFA* monounsaturated fatty acids, *SFA* saturated fatty acids, *PUFA* polyunsaturated fatty acids

^a^Calculated using robust linear regression

^†^Adjusted for age, sex and weight change

During the pre-surgical LED phase in trial 1 and during the lifestyle-intervention in trial 2, plasma levels of all saturated FAs decreased or remained unchanged (Table 2).

#### Monounsaturated fatty acids

In trial 1, plasma levels of total monounsaturated FAs, mainly oleic acid (C18:1 n-9), did not change after surgery (week 7–59) or after the 59-week lifestyle-intervention, while plasma levels of palmitoleic acid (C16:1 n-7) decreased and eicosenoic acid (C20:1 n-9) levels increased in both groups (Table 2). Vaccenic acid (C18:1 n-7 cis) levels decreased after surgery and remained unchanged in the lifestyle-group, resulting in a significant between-group difference (Table 3).

During the pre-surgical LED phase in trial 1 and during the lifestyle-intervention in trial 2 (lifestyle-cohort), plasma levels of total monounsaturated FAs, oleic acid and eicosenoic acid did not change during follow-up, while plasma levels of palmitoleic acid decreased (Table 2). Vaccenic acid levels increased during the pre-surgery LED phase, but did not change during follow-up in trial 2.

### Polyunsaturated fatty acids

#### n-6 fatty acids

In trial 1, plasma levels of total n-6 FAs did not change after surgery (week 7–59) or after the 59-week lifestyle-intervention (Table 2). By contrast, plasma levels of linoleic acid (LA; C18:2 n-6), eicosadienoic acid (EDA; C20:2 n-6) and dihomo-gamma-linolenic acid (DGLA; C20:3 n-6) increased after surgery, but did not change in the lifestyle-group. Gamma-linolenic acid (GLA; C18:3 n-6) increased in the surgery-group and decreased in the lifestyle-group, while arachidonic acid (AA; C20:4 n-6) levels decreased in the surgery-group and increased in the lifestyle-group. Plasma levels of GLA, EDA, DGLA and AA changed more after surgery than in the lifestyle group (Table 3).

During the pre-surgical LED-phase in trial 1 and during the lifestyle-intervention in trial 2 (lifestyle-cohort), plasma levels of total n-6 FAs and AA increased, while DGLA decreased (Table 2). Plasma levels of LA and EDA remained unchanged in the pre-surgical LED-phase, but increased in trail 2, while GLA levels decreased in the pre-surgical LED-phase, and did not change in trail 2.

#### n-3 fatty acids

In trial 1, plasma levels of total n-3 FAs, eicosapentaenoic acid (EPA; C20:5 n-3) and docosahexaenoic acid (DHA; C22:6 n-3) decreased after surgery (week 7–59), but increased in the lifestyle-group (Table 2). Alpha linolenic acid (ALA; C18:3 n-3) levels did not change during follow-up in the surgery-group, but decreased in the lifestyle-group, while docosapentaenoic (DPA; C22:5 n-3) levels increased in both groups (Table 2). Between-group differences in change were significant for ALA, DPA, EPA and DHA (Table 3).

During the pre-surgical LED-phase in trial 1, plasma levels of ALA decreased while plasma levels of all other n-3 FAs increased. During the lifestyle-intervention in trial 2, plasma levels of n-3 FAs did not change substantially.

### Associations between plasma levels of Lp(a) and fatty acids

In the surgery-group (weeks 7–59), changes in plasma Lp(a) levels were inversely associated with changes in plasma levels of total saturated FAs and palmitic acid, and positively associated with changes in plasma levels total n-6 FAs and LA (Table 4). In the lifestyle-group of trial 1, changes in Lp(a) levels were inversely associated with changes in levels of total saturated FAs, palmitic acid and stearic acid, and positively associated with changes in plasma levels of total n-6 FAs, LA, AA and DHA. In the pre-surgical LED-phase (baseline to week 7) there were inverse associations between changes in plasma levels of Lp(a) and total monounsaturated FAs, oleic acid, and positive associations with total n-3 polyunsaturated FAs and DHA. With respect to the ratio of Lp(a) and fatty acids: There were no significant differences in the ratio of Lp(a) and total or individual n-3 and n-6 fatty acids between the lifestyle- and surgery-group, at any timepoint (data not shown). In trial 2 (lifestyle-cohort), there was a positive association between changes in plasma levels of Lp(a) and changes in plasma levels of oleic acid.

**Table 4:** Associations between changes in plasma levels of fatty acids and changes in plasma levels of Lp(a)

	**Trial 1**						**Trial 2**	
	**Surgery-group**				**Lifestyle-group**		**Lifestyle-cohort**	
	Baseline—7 weeks (pre-surgery LED phase)		7 weeks—59 weeks (post-surgery phase)		Baseline—59 weeks		Baseline—20 weeks	
	_**Beta**_^a^	***P***	_**Beta**_^a^	***P***	_**Beta**_^a^	***P***	_**Beta**_^a^	***P***
**SFAs, % of total FA**								
Total SFA	-0.84 (-1.95, 0.27)	0.14	-3.61 (-5.75, -1.47)	**0.001**	-1.49 (-2.27, -0.72)	**< 0.001**	-1.51 (-3.47, 0.45)	0.13
Myristic acid, 14:0	-4.41 (-9.95, 1.13)	0.12	-2.81 (-12.7, 7.05)	0.57	-3.94 (-8.36, 0.47)	0.08	-7.67 (-17.6, 2.29)	0.13
Pentadecylic acid, 15:0	16.2 (-10.7, 43.0)	0.24	-4.88 (-52.9, 43.1)	0.84	5.13 (-22.0, 32.2)	0.71	-41.0 (-96.9, 14.9)	0.15
Palmitic acid, 16:0	-0.50 (-1.72, 0.72)	0.41	-3.65 (-5.72, -1.58)	**0.001**	-1.55 (-2.60, -0.50)	**0.004**	-0.89 (-3.30, 1.52)	0.47
Stearic acid, C18:0	-1.63 (-4.42, 1.15)	0.25	0.16 (-3.55, 3.87)	0.93	-2.13 (-4.22, -0.05)	**0.045**	-5.17 (-11.2, 0.83)	0.09
**MUFAs, % of total FA**								
Total MUFA	-0.81 (-1.38, -0.22)	**0.007**	-0.96 (-2.15, 0.22)	0.11	-0.44 (-0.98, 0.11)	0.12	1.25 (-0.00, 2.50)	0.05
Palmitoleic acid, 16:1 n-7	-1.49 (-4.43, 1.45)	0.32	-2.72 (-8.02, 2.58)	0.31	-2.71 (-5.43, 0.01)	0.05	-3.05 (-10.3, 4.23)	0.41
Oleic acid, 18:1 n-9	-0.90 (-1.59, -0.20)	**0.012**	-1.25 (-2.70, 0.20)	0.09	-0.51 (-1.14, 0.12)	0.11	1.67 (0.28, 3.06)	**0.019**
Vaccenic acid, 18:1 n-7 cis	4.79 (-2.62, 12.2)	0.20	-4.11 (-16.2, 7.93)	0.50	7.22 (-0.14, 14.6)	0.05	7.73 (-7.40, 22.9)	0.31
Eicosenoic acid, 20:1 n-9	1.57 (-29.3, 32.4)	0.92	6.63 (-37.2, 50.5)	0.76	4.07 (-22.5, 30.7)	0.76	35.0 (-40.6, 110.5)	0.36
**n-6 PUFAs, % of total FA**								
Total n-6 PUFAs	0.41 (-0.05, 0.88)	0.08	1.51 (0.66, 2.35)	**0.001**	0.46 (0.08, 0.84)	**0.019**	0.04 (-1.09, 1.16)	0.95
LA, C18:2 n-6	0.21 (-0.32, 0.75)	0.43	1.37 (0.42, 2.32)	**0.005**	0.49 (0.12, 0.86)	**0.011**	-0.69 (-1.97, 0.60)	0.29
GLA, C18:3 n-6	-6.66 (-19.2, 5.84)	0.10	1.23 (-18.6, 21.1)	0.90	-4.60 (-16.0, 6.79)	0.42	-11.9 (-35.4, 11.6)	0.32
EDA, C20:2 n-6	-3.68 (-48.7, 41.4)	0.87	5.55 (-50.7, 61.8)	0.85	-30.4 (-65.1, 4.30)	0.09	36.9 (-44.9, 118.6)	0.37
DGLA, C20:3 n-6	0.98 (-4.27, 6.23)	0.71	-7.38 (-11.1, -3.70)	0.71	0.63 (-3.98, 5.23)	0.79	7.61 (-6.11, 21.3)	0.28
AA, C20:4 n-6	0.92 (-0.21, 2.06)	0.11	0.24 (-1.58, 2.05)	0.80	1.70 (0.24, 3.16)	**0.023**	1.91 (-1.06, 4.89)	0.21
**n-3 PUFAs, % of total FA**								
Total n-3 PUFAs	2.43 (0.45, 4.42)	**0.017**	-0.27 (-2.14, 1.60)	0.77	0.75 (-0.61, 2.11)	0.28	-0.92 (-3.84, 2.01)	0.54
ALA, C18:3 n-3	-10.9 (-23.7, 1.99)	0.10	5.65 (-12.3, 23.6)	0.53	-1.68 (-11.4, 8.07)	0.73	3.91 (-12.1, 19.9)	0.63
EPA, C20:5 n-3	4.90 (0.83, 8.97)	0.02	-0.26 (-3.80, 3.27)	0.88	0.67 (-1.48, 2.82)	0.54	-2.18 (-8.54, 4.17)	0.50
DPA, C22:5 n-3	10.1 8–9.51, 29.8)	0.31	-0.68 (-24.1, 22.7)	0.95	4.77 (-9.94, 19.5)	0.52	-10.5 (-53.6, 32.6)	0.63
DHA, C22:6 n-3	3.85 (0.46, 7.24)	**0.026**	-1.16 (-5.15, 2.82)	0.56	**3.51 (0.01, 7.00)**	**0.049**	-2.06 (-7.81, 3.69)	0.48

*Abbreviations*: *AA* arachidonic acid, *ALA* alpha linolenic acid, *DGLA* dihomo-gamma-linolenic acid, *DHA* docosahexaenoic acid, *DPA* docosapentaenoic, *EDA* eicosadienoic acid, *EPA* eicosapentaenoic acid, *GLA* gamma-linolenic acid, *LA* linoleic acid, *MUFA* monounsaturated fatty acids, *SFA* saturated fatty acids, *PUFA* polyunsaturated fatty acids

^a^Calculated using robust linear regression

### Mediation analyses

Mediation analyses were performed based on data from trial 1. The association between the interventions (surgery vs. lifestyle) and changes in plasma levels of Lp(a) was not explained by changes in any of the individual FAs or groups of FAs, neither when comparing changes in plasma Lp(a) levels and FAs in the post-surgical phase (7 weeks to 59 weeks) with the lifestyle group (Supplementary table 1), nor when comparing the surgery group (baseline to 59 weeks) with the lifestyle group (data not shown).

## Discussion

This study shows that, in people with overweight and obesity, bariatric surgery was associated with reduced plasma Lp(a) levels, whereas lifestyle interventions including calorie restriction were associated with increased plasma Lp(a) levels. Bariatric surgery and lifestyle interventions also differentially influenced plasma levels of FAs: Plasma levels of total saturated FAs remained unchanged after surgery, but decreased after lifestyle interventions. Also, plasma levels of the n-6 FA AA and total n-3 FAs decreased after surgery, but increased after lifestyle interventions. However, there was no evidence of FAs mediating the differential effects of the interventions (surgery vs. lifestyle) on plasma Lp(a) levels.

### Comparisons with other studies—what does the current work add to the existing knowledge

RYGB is associated with reduced cardiovascular disease risk [24] and reduced risk of all-cause mortality [25], while lifestyle modifications tend to have less influence on morbidity and mortality [26]. The effects of RYGB on decreased cardiovascular disease risk and mortality are thought to mainly be driven by weight loss. However, a reduction in circulating levels of Lp(a) may also add to the beneficial effects of bariatric surgery on cardiovascular disease risk. The Lp(a) lowering effect of RYGB, observed in this study, is in accordance with results from previous studies. A recent meta-analysis including 13 studies and 1551 adults and adolescents revealed a significant decrease in circulating Lp(a) following different types of bariatric surgery (standardized mean difference; -0.438, 95% CI: -0.702, -0.174) [8]. The heterogeneity between the studies was, however, large, and the mean (SD) Lp(a) levels at baseline ranged from 14.0 (3.65) to 258.2 (378) nmol/L. Meta-regression showed that there were no associations between changes in Lp(a) levels and BMI change or duration of follow-up. Further, in line with the present results, in a study involving 60 females, with and without overweight, a 9% increase in Lp(a) levels was shown after a 6-month period of calorie restriction [27]. Also, in a cohort overlapping with the trial 2 cohort, Lp(a) increased in patients with overweight, with and without T2D, undergoing a calorie restricted diet for 3–4 months [7]. However, other studies have shown no change in Lp(a) levels after various dietary interventions aimed at weight loss [28–30].

Bariatric surgery and lifestyle-interventions did also differently influence FA levels. After bariatric surgery, plasma levels of total saturated FAs did not change from baseline, however plasma levels of palmitic acid decreased, while myristic acid, pentadecylic acid and stearic acid increased. The increased proportions of the saturated FAs myristic acid, pentadecylic acid and stearic acid following RYGB have previously been shown [31]. Total plasma saturated FAs decreased after the lifestyle interventions, which was mainly due to a reduction in palmitic acid. This finding is in accordance with results from a previous 12-week randomized controlled trial comparing mild-calorie-restriction (minus 300 kcal/day) with a control diet [15]. Calorie restricted diets typically include low levels of total fat and, in particular, saturated fats, as was also the case for the participants in the lifestyle intervention groups in this study. However, dietary intake of saturated and also mono-unsaturated fats may not necessarily correlate with plasma levels as these dietary FAs are endogenously synthesized and remodeled [32]. Plasma levels of the polyunsaturated FAs, on the other hand, correlate more strongly with dietary intake, and may better reflect dietary intake. Plasma levels of a number of n-3 FAs as well as the n-6 FA AA decreased after surgery, but increased during lifestyle interventions. Previous studies on the effect of calorie restriction and bariatric surgery on polyunsaturated FA levels showed somewhat conflicting results. In patients undergoing RYGB, the proportions of circulating n-3 [33] and n-6 FAs [16] increased from baseline to 1 year after surgery. In contrast, among 13 women undergoing RYGB, phospholipid FA composition was similar to baseline levels at 6 months post-surgery, except for a decrease in content of EPA [17]. Mild caloric restriction (minus 300 kcal/day) in 80 patients with overweight, resulted in greater reductions in plasma levels of some n-3 and n-6 FAs compared to control diet [15]. Unfortunately, data on dietary intake was not collected in the current study, neither in the lifestyle intervention groups nor in the bariatric surgery group. Thus, the strength of the relationship between changes in plasma FA levels and dietary intake cannot be assessed in the present study. However, one could speculate that the reduced intake of FAs in patients undergoing calorie restrictive diets has a different effect on circulating FAs and lipid metabolism compared with patients undergoing bariatric surgery where there is reduced absorption of FAs [34], changes in the composition of microbiota and often altered dietary preferences [35].

The hypothesis of the present study was that bariatric surgery and lifestyle interventions would differently influence Lp(a) levels through a dissimilar effect on plasma FA levels. In the surgery-group, changes in Lp(a) were inversely associated with changes in saturated FAs and positively with changes in n-6 FAs, but there was no consistent pattern of associations among the different lifestyle intervention groups. An increased intake of total and saturated fats has been reported to decrease Lp(a) levels [12, 36]. However, mediation analysis failed to show an important role for any of the saturated FAs, in mediating the effect of surgery versus lifestyle intervention on Lp(a) levels in trial 1, an important result the current study adds to the existent knowledge. It has previously been shown that plasma levels of AA were positively associated with Lp(a) levels in patients suffering from familial hypercholesterolemia [9]. AA is an antagonist of the farnesoid X receptor (FXR) [37], and FXR activation has been found to decrease Lp(a) levels [38]. Thus the increased levels of AA during the lifestyle interventions in the present study, could potentially have caused the observed increase in Lp(a) levels, whereas the decreased AA levels after surgery may have resulted in the observed reduced Lp(a) levels. However, according to the mediation analyses, the different effects of surgery and lifestyle interventions on Lp(a) levels were not explained by changes in plasma levels of AA, nor of any other FA or FA category. Bile acids also act as FXR agonists. Although bile acids were not measured in this study, previous studies have shown that circulating levels of bile acids are increased after RYGB [39, 40], which may partly explain the lowering of Lp(a) levels among the surgical patients. Interestingly, bile acid synthesis and levels have been shown to be increased in women with obesity, and to be normalized within 3 days on a caloric restriction diet [41]. Diet-induced lowering of bile acid production may therefore partly explain the increased Lp(a) levels observed during the lifestyle interventions. Future studies should determine whether the differential effects of surgery and lifestyle intervention on Lp(a) are mediated by changes in bile acid levels. Furthermore, one could speculate that exercise may have influenced the observed change in Lp(a) levels. Results from studies on the effect of exercise on Lp(a) levels have been inconsistent, with some reporting no effect while others have reported mildly increased or decreased levels [42]. However, studies among younger individuals or patients with diabetes, showed moderate Lp(a)-lowering effects by exercise. In trial 1, the participants underwent a physical activity intervention, and the majority of the patients reported that they completed > 3 h of light physical activity per week and > 3 h of vigorous physical activity per week during follow-up. Participants in the surgery group did not follow an exercise program prior to or following surgery. Exercise was also encouraged in trial 2, but the amount of physical activity performed did not change significantly from baseline. As Lp(a) increased during all lifestyle interventions even though only participants in the lifestyle-group of trail 1 followed an exercise program, it is less likely that the observed increase in Lp(a) was caused by exercise.

## Strenghts and limitations

The strengths of the present study are its prospective design and the use of two independent trials with a relatively high number of patients with detailed analyses on both plasma FAs and Lp(a). Limitations include the non-randomized design, and plasma levels of Lp(a) and FAs being exploratory endpoints in both trials. Further, as the participants had been referred to a tertiary care center, these findings may not be generalized to all individuals with overweight and obesity. Of note, plasma Lp(a) levels were higher in trial 2 compared with trial 1, which may be partly explained by differences in analytical methods between the trials. Plasma samples from trial 2 were measured using a particle-enhanced immunoturbidimetric method by DiaSys Diagnostic System, but also later re-analyzed in a sub-group of patients using Roche Diagnostics, as applied in trial 1. The median Lp(a) value was 13% higher using the DiaSys Diagnostic System versus using the Roche Diagnostics method. Repeated freezing/thawing cycles may influence Lp(a) levels in samples [43]. However, the plasma samples were only frozen and thawed once before Lp(a) analyses in both trials, thus this is likely not an issue here. Another possible explanation may be differences in ethnicities between the trials. More than 55% of the participants in trial 2 were of non-White ethnicity, whereas 98% of the participants in trial 1 were White. Lp(a) levels are reported to vary across ethnicities, and people of non-White ethnicities are reported to have higher Lp(a) levels compared with those of White ethnicity [42]. All participants in trial 2 had T2D compared with only 20% in trial 1. Previous studies have shown conflicting results regarding whether patients with T2D having higher or lower plasma Lp(a) levels than patients without T2D [7, 44, 45]. Also, the participants in trial 2 were older than the participants in trial 1, and some, but not all, studies suggest that Lp(a) increases with age [46–50]. Polyunsaturated FAs are also susceptible to degradation through freezing/thawing cycles. In trial 2, the samples were frozen only once prior to the FA analysis, while in trial 1, the samples were frozen twice before analysis. There is thus a possibility that there may have been some degradation of the polyunsaturated FAs in trial 1.

## Conclusion

Lp(a) levels decreased in patients with obesity who underwent RYGB, but increased in patients with overweight or obesity undergoing lifestyle interventions. The mechanisms behind the Lp(a) lowering effect of bariatric surgery are unknown. The results of this study indicate that alterations of plasma levels of different FAs following RYGB do not explain the changes in circulating Lp(a) levels. In addition, changes in Lp(a) levels were not explained by changes in bodyweight. As individuals with obesity have an increased risk of cardiovascular disease, which is reduced after RYGB, one could speculate whether the reduction in circulating levels of Lp(a) partly explains the beneficial effects of RYGB on cardiovascular disease risk is at least partly explained by the reduction in circulating levels of Lp(a). On the other hand, the increase in Lp(a) seen after lifestyle interventions may increase cardiovascular risk in individuals with obesity. If the mechanisms behind the increase in Lp(a) by lifestyle interventions were known, it becomes possible to take targeted actions to reduce this potentially negative side effect. Our results make it less likely that manipulating fatty acid composition will contribute to the solution. Thus future studies should clarify the mechanisms underlying the decrease in Lp(a) levels after RYGB and the increase in Lp(a) levels following lifestyle interventions. Long-term follow-up studies are also required to determine whether elevated Lp(a) levels, observed after energy restricted diets, are associated with an increased incidence of cardiovascular disease in patients with overweight and obesity.

## Supplementary Information

**Additional file 1:** **Table S1.** The mediating effect of fatty acids on the relationshipbetween group (surgery vs. lifestyle) and plasma levels of Lp(a).

**Additional file 2.**

## Data Availability

Access to data collected from this study, including de-identified individual-participant data, will be made available following publication upon e-mail request to the corresponding author (HB). After approval of a proposal, data will be shared with investigators whose proposed use of the data is in accordance with the consent given by the participants and in accordance with Norwegian and/or Dutch laws and legislations.

## Abbreviations

AA: Arachidonic acid
ALA: Alpha linolenic acid
BMI: Body mass index
DGLA: Dihomo-gamma-linolenic acid
DHA: Docosahexaenoic acid
DPA: Docosapentaenoic
EDA: Eicosadienoic acid
EPA: Eicosapentaenoic acid
FA: Fatty acid
GLA: Gamma-linolenic acid
HDL: High density lipoprotein
IQR: Interquartile range
LA: Linoleic acid
LDL: Low density lipoprotein
LED: Low energy diet
Lp(a): Lipoprotein (a)
RDA: Recommended Dietary Allowance
RYGB: Roux-en-Y gastric bypass
SD: Standard deviation
TBWL: Total body weight loss
T2DM: Type 2 diabetes mellitus
