# Supplementary material for: Differential effects of bariatric surgery and lifestyle interventions on plasma levels of Lp(a) and fatty acids
Source: Lipids Health Dis. 2022 Dec 28;21:145. doi: 10.1186/s12944-022-01756-1 (PMC9795629; doi:10.1186/s12944-022-01756-1)
Supplement: Supplementary file 1 — Additional file 1: Table S1. The mediating effect of fatty acids on the relationshipbetween group (surgery vs. lifestyle) and plasma levels of Lp(a). [file 12944_2022_1756_MOESM1_ESM.docx]

| **Table S1. The mediating effect of fatty acids on the relationship between group (surgery vs. lifestyle) and plasma levels of Lp(a)** | | |
| --- | --- | --- |
| **Mediation variable** | **Indirect effect (95% CI)** | **Mediation effect (*P*-value)^*^** |
|  |  |  |
| **SFAs, % of total fatty acids** |  |  |
| Total SFA | -0.85 (-2.14 to 0.44) | 0.29 |
| Myristic acid, 14:0 | 1.55 (-0.92 to 4.57) | 0.38 |
| Pentadecylic acid, 15:0 | 1.14 (-1.10 to 4.09) | 0.31 |
| Palmitic acid, 16:0 | 0.85 (-0.19 to 2.53) | 0.23 |
| Stearic acid, C18:0 | 0.23 (-3.34 to 3.84) | 0.89 |
| **MUFAs, % of total fatty acids** |  |  |
| Total MUFA | 0.02 (-0.58 to 1.12) | 0.90 |
| Palmitoleic acid, 16:1 n-7 | -0.07 (-0.98 to 0.73) | 0.83 |
| Oleic acid, 18:1 n-9 | -0.25 (-2.60 to 2.15) | 0.95 |
| Vaccenic acid, 18:1 n-7 cis | -0.25 (-2.54 to 2.18) | 0.81 |
| Eicosenoic acid, 20:1 n-9 | -0.27 (-1.52 to 0.57) | 0.55 |
| **n-6 PUFAs, % of total fatty acids** |  |  |
| Total n-6 PUFAs | 0.02 (-0.54 to 0.89) | 0.92 |
| LA, C18:2 n-6 | -0.12 (-1.02 to 0.71) | 0.72 |
| GLA, C18:3 n-6 | 0.96 (-0.57 to 3.14) | 0.36 |
| EDA, C20:2 n-6 | -1.37 (-4.25 to 1.29) | 0.36 |
| DGLA, C20:3 n-6 | 1.92 (-0.07 to 4.39) | 0.15 |
| AA, C20:4 n-6 | -0.40 (-3.74 to 2.78) | 0.82 |
| ***n-3 PUFAs, % of total fatty acids*** |  |  |
| Total n-3 PUFAs | -1.04 (-3.44 to 1.10) | 0.42 |
| ALA, C18:3 n-3 | 0.63 (-1.05 to 2.57) | 0.48 |
| EPA, C20:5 n-3 | -1.10 (-3.30 to 0.58) | 0.27 |
| DPA, C22:5 n-3 | 0.32 (-0.86 to 2.00) | 0.59 |
| DHA, C22:6 n-3 | -0.24 (-3.59 to 2.96) | 0.91 |
| Abbreviations: AA; arachidonic acid, ALA; alpha linolenic acid, CI; confidence interval, DGLA; dihomo-gamma-linolenic acid, DHA; docosahexaenoic acid, DPA; docosapentaenoic, EDA; eicosadienoic acid, EPA; eicosapentaenoic acid, GLA; gamma-linolenic acid, LA; linoleic acid, MUFA; monounsaturated fatty acids, SFA; saturated fatty acids, PUFA; polyunsaturated fatty acids. *Calculated using Sobel test (calculates whether the indirect effect of the independent variable (group) on the dependent variable (delta Lp(a)) through the mediator variable (fatty acid) is significant. Changes in plasma Lp(a) levels and fatty acids in the surgery-group (post-surgery phase) (7 weeks to 59 weeks) were compared with changes in plasma Lp(a) levels and fatty acidss in the lifestyle group (baseline-59 weeks). | | |
