# Supplementary material for: Differential effects of bariatric surgery and lifestyle interventions on plasma levels of Lp(a) and fatty acids
Source: Lipids Health Dis. 2022 Dec 28;21:145. doi: 10.1186/s12944-022-01756-1 (PMC9795629; doi:10.1186/s12944-022-01756-1)
Supplement: Supplementary file 2 — Additional file 2. [file 12944_2022_1756_MOESM2_ESM.pdf]

Manuscript version 3

By: Heidi Borgeraas

As of: Nov 30, 2022 7:23:56 AM
5,059 words - 32 matches - 19 sources

Similarity Index

8%

Mode: Similarity Report

paper text:

Abstract Background Limited evidence suggests that surgical and non-surgical obesity treatment differentially influence plasma Lipoprotein (a) [Lp(a)] levels. Further, a novel association between plasma arachidonic acid and Lp(a) has recently been shown, suggesting that fatty acids are a possible target to influence Lp(a). Here, the effects of bariatric surgery and lifestyle interventions on plasma levels of Lp(a) were compared, and it was examined whether the effects were mediated by changes in plasma fatty acid (FA) levels. Methods The study includes two independent trials of patients with overweight or obesity. Trial 1: Two-armed intervention study including 82 patients who underwent a 7-week low energy diet (LED), followed by Roux-en-Y gastric bypass and 52-week follow-up (surgery- group), and 77 patients who underwent a 59-week energy restricted diet- and exercise- program (lifestyle-group). Trial 2: A clinical study including 134 patients who underwent a 20-week very-LED/LED (lifestyle-cohort). Results In the surgery-group, Lp(a) levels [median (interquartile range)] tended to increase in the pre-surgical LED-phase [17(7-68)-21(7-81)nmol/L, p=0.05], but decreased by 48% after surgery [21(7-81) - 11(7-56)nmol/L, p<0.001]. In the lifestyle-group and lifestyle-cohort, Lp(a) increased by 36%[14(7-77) - 19(7-94)nmol/L, p<0.001] and 14%[50(14-160) - 57(19- 208)nmol/L, p<0.001], respectively. Changes in Lp(a) were independent of weight loss. Plasma levels of total saturated FAs remained unchanged after surgery, but decreased after lifestyle interventions. Arachidonic acid and total n-3 FAs decreased after surgery, but increased after lifestyle interventions. Plasma FAs did not mediate the effects on Lp(a). Conclusion Bariatric surgery reduced, whereas lifestyle interventions increased plasma Lp(a), independent of weight loss. The interventions differentially influenced changes in plasma FAs, but these changes did not mediate changes in Lp(a). 25

Introduction Lipoprotein (a) [(Lp(a)] is a low density lipoprotein (LDL)-like particle with an apolipoprotein (a) [**apo(a)] attached to** 4

the ApoB100, and is considered an independent risk factor for cardiovascular disease(1-4). Plasma Lp(a) levels can be manipulated, and studies indicate that as much as

25% of the variance in Lp(a) levels is ascribed **to lifestyle** 1

factors(5). However, the mechanisms by which Lp(a) levels are regulated are not well understood. Lp(a) levels are influenced by caloric restriction and bariatric surgery, but do not appear to be regulated by weight loss per se(6). It has previously been shown that weight loss after energy- restricted dieting was associated with

an increase in plasma Lp(a) levels in adults with or without type 2 diabetes (T2D), while 1

plasma Lp(a) levels showed a strong tendency to decrease in patients without T2D who underwent bariatric surgery(7). A recent meta analysis showed that bariatric surgery significantly decreased circulating Lp(a) levels, and that the decrease in Lp(a) was not associated with change in body mass index (BMI)(8). Lp(a) levels may also be regulated, to some extent, by changes in plasma fatty acids (FAs). A positive, novel association between plasma levels of the n-6 FA arachidonic acid (AA) and Lp(a) in patients with familial hypercholesterolemia has recently been shown(9). Other studies have shown that an increased intake of total- and saturated fat is accompanied by a decrease in Lp(a) levels(10-13), and that supplementation of conjugated LA lead to increased Lp(a) levels(14). The composition of the plasma FA pool may be altered both by caloric restriction(15) and bariatric surgery(16-18), where possible contributing factors are the reduced dietary intake, changes in the dietary composition, malabsorption of lipids(19) and release of FAs from the body fat deposits during weight loss. Whether changes in plasma Lp(a) levels following caloric restriction or bariatric surgery are mediated by changes in plasma FA levels is not known. It is important to identify opportunities to reduce adverse changes to Lp(a) during weight loss dieting, through intervening on plasma FAs. In this study, the

effects of Roux-en-Y gastric bypass surgery (RYGB) and an intensive lifestyle intervention 2

, including caloric restriction and exercise, on plasma Lp(a) and FA levels in patients with obesity were compared, and it was also examined whether possible effects on plasma Lp(a) levels were mediated by changes in plasma FA levels. The effects of a lifestyle intervention on plasma Lp(a) and FA levels in an independent cohort of patients with T2D and overweight or obesity were also examined. Methods Study subjects and design This study includes two independent trials. Trial 1 is a two-armed non-randomized study which compared the 1-year effects of RYGB (surgery-group) with intensive lifestyle intervention (lifestyle-group) (Clinicaltrials.gov NCT00626964), conducted at the Morbid Obesity Centre, Vestfold Hospital Trust, Tønsberg, Norway between February 2008 and February 2011. Inclusion criteria were BMI ≥ 40 kg/m2, or ≥ 35 kg/m2 and at least one obesity related comorbidity. The primary outcome (arterial stiffness) and data on weight-loss and changes in metabolic biomarkers have previously been published(20, 21). Trial 2 includes individuals who participated in the Prevention Of Weight Regain (POWER) cohort study [Netherlands Trial Register NL2140 (NTR2264)] (lifestyle-cohort)(22). Participants were recruited at the outpatient diabetes clinic of the Erasmus University Medical Centre, Rotterdam, The Netherlands, between March 2010 and April 2015. The inclusion criteria were BMI >27 kg/m² and T2D. The primary outcome (Lp(a) levels) and data on weight-loss and changes in metabolic biomarkers have previously been published(7). Trial 1 was approved by the The Regional Committees for Medical and Health Research Ethics in Norway (code: S-05175) and trial 2 was approved by the Medical Ethics Committee of the Erasmus Medical Center (reference numbers MEC-2009-143, MEC-2014-090 and MEC 2016-604). Both trials were conducted according to the principles in the Declaration of Helsinki, and written informed consent was provided by all the participants. Interventions Trial 1: The participants in the surgery-group followed a low energy diet (LED) (<900 kcal per day) for 7 weeks prior to surgery (pre-surgery phase), and were followed for 52 weeks after surgery (post-surgery

phase) where they received standard follow-up care at the Morbid Obesity Centre - a total follow-up of 59 weeks. The participants in the lifestyle-group underwent a dietary and physical activity intervention which lasted for a total of 59 weeks(20, 21). They received nutritional counseling according to Norwegian nutritional guidelines and every participant's energy intake was reduced by 1000 kcal/day, they also underwent 90 min supervised training sessions, including weight bearing and aerobic exercise, 3 days/week during the first 12 weeks. Thereafter, the participants received monthly follow-ups, and

were advised to maintain physical activity for 60-90 min per day 2

throughout the study period (59 weeks). Trial 2: The participants underwent a dietary intervention which lasted for a total of 20 weeks. During the first 8 weeks, the participants followed a very LED of approximately

750 kcal per day , which consisted **of two meal replacements (Glucerna SR, Abbott Nutrition, Lake Forest, Illinois, USA**), plus **a small dinner** 1

, providing a total of 67 g carbohydrates, 11.5 g of fibre,

54 g protein and 32 g fat (of which 16 g monounsaturated FAs) daily **and micronutrients** 1

according to RDA recommendations.

Thereafter, energy intake was slowly increased up to approximately 1300 **kcal per day** 1

over 12 weeks. In addition, 30-60 minutes of daily exercise was encouraged during the entire intervention. Outcomes The main outcomes were plasma levels of Lp(a) and FAs. Plasma levels were measured at baseline (trial 1 and 2), 7 and 59 weeks (trial 1), and at 20 weeks (trial 2). Laboratory analyses In trial 1, plasma Lp(a) concentrations were measured using a particle-enhanced immunoturbidimetric method, by Roche Diagnostics at an accredited medical laboratory, Oslo University Hospital, Rikshospitalet, Oslo, Norway (NS-EN ISO 15189:2007). The samples were stored for 6-9 years at -80?C, and had not been thawed prior to the Lp(a) analysis. In trial 2 Lp(a) concentrations were measured using the Diagnostic System #171399910930 7 (DiaSys Diagnostic System, GmbH, Holzheim, Germany). The samples were stored for 5-10 years at -80?C before analysis, and had not been thawed prior to the Lp(a) analysis. Plasma Lp(a) levels were subsequently re-measured in a sub-group of participants from trial 2 using Roche Diagnostics to evaluate method agreement. Measurements of fasting serum blood glucose and lipoprotein profiles have been described previously(21, 22). Plasma free FA profiles were determined

by Gas Chromatography-Flame Ionization Detector analysis at the commercial laboratory Vitas Analytical Services 3

. The serum samples were thawed and aliquoted to dried blood spot (DBS) paper (Whatman 903 paper) until GC-analysis. One 4.7 mm punch of human plasma DBS paper were methylated with sodium methoxide in methanol. After methylation, FA methyl esters (FAME) were extracted with hexane.

After thorough mixing and centrifugation , 3 µl **of the** hexane **phase was injected into the** 11

GC-FID. GC-FID was performed with an Agilent 7890A Gas Chromatograph System 9

(

Agilent Technologies, Palo Alto, CA , USA). **Separations** was **performed on a SP-2380 (30 m × 0.25 mm i.d. × 0.25 µm film thickness) column from Supelco** 5

. The results are shown as percentages of total FAs. In trail 1, the samples were stored for 7-10 years at -80?C, and were frozen twice before FA analysis. In trial 2, the samples were stored for 5-10 years at -80?C and had not been thawed prior to FA analysis. Statistical analyses Data

are presented as means [standard deviation (SD)] or medians [interquartile range (IQR)] for continuous data, and as counts (%) **for categorical data** 7

.

McNemar's test, paired T-test or **Wilcoxon Signed Rank test were** 14

used when investigating within-group changes. Statistical between-group comparisons were made between the lifestyle-group of trail 1 (baseline-59 weeks) and the surgery-group of trial 1 (week 7-59). Between-group differences in changes from baseline (lifestyle-group) and from week 7 (surgery-group) to end of 8 intervention were estimated using a robust linear regression approach which is a non- parametric iterative method using weights from absolute residuals. The results are expressed as means (95% CI), and STATA version 15.0 was used to perform the analyses.

Mediation analyses were performed using the PROCESS macro (version 3.3) for SPSS written **by** 8
A **F Hayes**

(23), with group (lifestyle vs. surgery) as the independent variable, change in Lp(a) level (7 weeks to 59 weeks for the surgery-group and baseline to 59 weeks for the lifestyle- group) as the dependent variable and change in FA level (7 weeks to 59 weeks for the surgery-group and baseline to 59 weeks for the lifestyle-group) as the mediator variable. Hayes uses the three steps as originally suggested by Byron and Kenny.

In step 1 the independent variable is **regressed on the** mediator. **In step 2 the independent variable** is **regressed on the dependent variable. In step 3 the** final model with **the** 6

independent variable and the moderator as covariates is fitted and the proportion of the association between the independent and dependent variable which is explained by the mediator can be calculated. P- values in the mediation analyses were calculated using the Sobel test. P-values <0.05 were considered statistically significant. The analyses were considered exploratory, thus no corrections for multiple testing were performed. Results Characteristics of the participants In trial 1, 82 of the 98

patients in the surgery-group, and 77 **of** the 102 **patients in the lifestyle- group** 10

completed the 59-week follow-up, leaving 159 patients to be included in the present analysis (Figure 1). Trial 2 (lifestyle-cohort) included 161 participants, whereof the 134 participants who had measured plasma Lp(a) and FAs before and after the intervention were included in the current analysis. The baseline characteristics of participants in both trials are presented in Table 1. In trial 1, more than 60% (n=102) of the participants in both arms were female, and 97% (n=155) were White. The participants in the surgery-group were younger (41 years vs. 47

years, p=0 .011), **had a higher BMI (46 kg** /m2 **vs** 18

. 42 kg/m2, p<0.001), and were less often diagnosed with cardiovascular disease (2% vs. 16%, p=0.004), compared with participants in the lifestyle- group. A total of 26% (n=41) had T2D, and 19% (n=30) were prescribed a statin, with no difference between the groups. In trial 2, 60% (n=80) of the participants were women and 45% (n=73) were White, the median age was 55 years and the mean BMI 35 kg/m2. All participants were diagnosed with T2D, 49% (n=65) were on insulin treatment, 16% (n=22) were diagnosed with cardiovascular disease and 58% (n=77) received statin treatment. Weight loss and changes in metabolic biomarkers In trial 1, the initial 7-week LED in the surgery-group led to a mean (95% CI) total body weight loss (TBWL) of 7 (6-7)%, followed by an additional 27 (25-28)% TBWL after surgery (week 7-59) (Table 1). The lifestyle-group had a TBWL of 10 (8-12)% at 59-week follow-up. The participants in trial 2 (lifestyle-

cohort) had a TBWL of 9 (8-10)% at 20 weeks follow-up. The serum levels of triglycerides, fasting glucose and C-reactive protein decreased significantly over time in both groups in trial 1 and also in trial 2 (Table 1). Serum total 10 cholesterol and LDL cholesterol remained unchanged in the lifestyle-group in trial 1, but decreased significantly after surgery in trial 1 and also in trial 2. High density lipoprotein (HDL) cholesterol levels increased in both groups in trial 1 and also in trial 2. Lipoprotein (a) In the surgery-group, the median (IQR) concentration of Lp(a) tended to increase during the 7-week pre-surgery LED-phase [from 17 (7-68) to 21 (7-81) nmol/L, p=0.05], but were decreased by 48% after surgery [from 21 (7-81) to 11 (7-56) nmol/L, p<0.001] at week 59 (Table 1).

There was also a significant 35% **decrease in plasma Lp(a) levels** 17

when comparing baseline values to values at 59 weeks [from 17 (7-68) to 11 (7-56) nmol/L, p=0.004] in the surgery-group. Median plasma levels of Lp(a) increased by 36% [from 14 (7-77) to 19 (7-94) nmol/L, p<0.001] during 59-week follow-up in the lifestyle-group (Trial 1). There was a significant difference in change [mean (95 % CI)] in Lp(a) levels when comparing the surgery-group (week 7-59) with the lifestyle-group (baseline-59 weeks) [-8.0 (-11.1, -4.8) nmol/L, p<0.001], and adjusting for changes in body weight, sex and age did not significantly influence the results (data not shown). Changes in Lp(a) levels from week 7 to week 59 for each individual participant in the surgery-group and from baseline to 59 weeks for the participants in the lifestyle-group, are presented in Figure 2A. The figure shows that the majority of participants in the surgery group experienced a reduction in plasma Lp(a) levels, while the majority of the participants in the lifestyle-group experienced increased plasma levels of Lp(a) during follow-up. In trial 2 (lifestyle-cohort), median plasma levels of Lp(a) increased by 14% [from 50 (14-160) to 57 (19-208) nmol/L, p<0.001] during 20-week follow- up. Changes in Lp(a) levels from baseline to 20 weeks for the participants in trial 2 are presented in Figure 2B.

There was no **significant** association **between change in** Lp(a) levels **and change in** body weight **in** 15

trial 2 (data not shown). Fatty acids Saturated fatty acids In trial 1, plasma levels of total saturated FAs did not change after surgery (week 7-59), but decreased slightly after the 59-week lifestyle-intervention (Table 2). Plasma levels of

myristic acid (C14:0), pentadecylic acid (C15:0) and **stearic acid (C18:0** 12

) increased after surgery, but decreased (myristic acid) or remained unchanged (pentadecylic acid and stearic acid) in the lifestyle-group. Palmitic acid (C16:0) levels decreased in both groups. Plasma levels of all the individual saturated FAs changed significantly more after surgery than after the lifestyle intervention (Table 3). During the pre-surgical LED phase in trial 1 and during the lifestyle-intervention in trial 2, plasma levels of all saturated FAs decreased or remained unchanged (Table 2). Monounsaturated fatty acids In trial 1, plasma levels of total monounsaturated FAs, mainly oleic acid (C18:1 n-9), did not change after surgery (week 7-59) or after the 59-week lifestyle-intervention, while plasma levels

of palmitoleic acid (C16:1 n-7) decreased and eicosenoic acid (C20:1 n-9) levels increased in both groups (Table 2). Vaccenic acid (C18:1 n-7 cis) levels decreased after surgery and remained unchanged in the lifestyle-group, resulting in a significant between- group difference (Table 3). During the pre-surgical LED phase in trial 1 and during the lifestyle-intervention in trial 2 (lifestyle-cohort), plasma levels of total monounsaturated FAs, oleic acid and eicosenoic acid did not change during follow-up, while plasma levels of palmitoleic acid decreased (Table 2). Vaccenic acid levels increased during the pre-surgery LED phase, but did not change during follow-up in trial 2. Polyunsaturated fatty acids n-6 fatty acids In trial 1, plasma levels of total n-6 FAs did not change after surgery (week 7-59) or after the 59-week lifestyle-intervention (Table 2). By contrast, plasma levels of linoleic acid (LA; C18:2 n-6), eicosadienoic acid (EDA; C20:2 n-6) and dihomo-gamma-linolenic acid (DGLA; C20:3 n-6) increased after surgery, but did not change in the lifestyle-group. Gamma-linolenic acid (GLA; C18:3 n-6) increased in the surgery-group and decreased in the lifestyle-group, while arachidonic acid (AA; C20:4 n-6) levels decreased in the surgery-group and increased in the lifestyle-group. Plasma levels of GLA, EDA, DGLA and AA changed more after surgery than in the lifestyle group (Table 3). During the pre-surgical LED-phase in trial 1 and during the lifestyle-intervention in trial 2 (lifestyle-cohort), plasma levels of total n-6 FAs and AA increased, while DGLA decreased (Table 2). Plasma levels of LA and EDA remained unchanged in the pre-surgical LED-phase, but increased in trail 2, while GLA levels decreased in the pre-surgical LED-phase, and did not change in trail 2. n-3 fatty acids In trial 1, plasma levels of total n-3 FAs, eicosapentaenoic acid (EPA; C20:5 n-3) and docosahexaenoic acid (DHA; C22:6 n-3) decreased after surgery (week 7-59), but increased in the lifestyle-group (Table 2). Alpha linolenic acid (ALA; C18:3 n-3) levels did not change during follow-up in the surgery-group, but decreased in the lifestyle-group, while docosapentaenoic (DPA; C22:5 n-3) levels increased in both groups (Table 2). Between- group differences in change were significant for ALA, DPA, EPA and DHA (Table 3). During the pre-surgical LED-phase in trial 1, plasma levels of ALA decreased while plasma levels of all other n-3 FAs increased. During the lifestyle-intervention in trial 2, plasma levels of n-3 FAs did not change substantially. 13 Associations between plasma levels of Lp(a) and fatty acids In the surgery-group (weeks 7-59), changes in plasma Lp(a) levels were inversely associated with changes in plasma levels of total saturated FAs and palmitic acid, and positively associated with changes in plasma levels total n-6 FAs and LA (Table 4). In the lifestyle- group of trial 1, changes in Lp(a) levels were inversely associated with changes in levels of total saturated FAs, palmitic acid and stearic acid, and positively associated with changes in plasma levels of total n-6 FAs, LA, AA and DHA. In the pre-surgical LED-phase (baseline to week 7) there were inverse associations between changes in plasma levels of Lp(a) and total monounsaturated FAs, oleic acid, and positive associations with total n-3 polyunsaturated FAs and DHA. In trial 2 (lifestyle-cohort), there was a positive association between changes in plasma levels of Lp(a) and changes in plasma levels of oleic acid. Mediation analyses Mediation analyses were performed based on data from trial 1. The association between the interventions (surgery vs. lifestyle) and changes in plasma levels of Lp(a) was not explained by changes in any of the individual FAs or groups of FAs, neither when comparing changes in plasma Lp(a) levels and FAs in the post-surgical phase (7 weeks to 59 weeks) with the lifestyle group (Supplementary table 1), nor when comparing the surgery group (baseline to 59 weeks) with the lifestyle group (data not shown). Discussion This study shows that, in people with overweight and obesity, bariatric surgery was associated with reduced plasma Lp(a) levels, whereas lifestyle interventions including caloric restriction were associated with increased plasma Lp(a) levels. Bariatric surgery and lifestyle interventions also differentially influenced plasma levels of FAs: Plasma levels of total saturated FAs remained unchanged after surgery, but decreased after lifestyle interventions. Also, plasma levels of the n-6 FA AA and total n-3 FAs decreased after surgery, but increased after lifestyle interventions. However, there was no evidence of FAs mediating the differential effects of the interventions

(surgery vs. lifestyle) on plasma Lp(a) levels. Comparisons with other studies and what does the current work add to the existing knowledge RYGB is associated with reduced cardiovascular disease risk(24) and reduced risk of all- cause mortality(25), while lifestyle modifications tend to have less influence on morbidity and mortality(26). The effects of RYGB on decreased cardiovascular disease risk and mortality are thought to mainly be driven by weight loss. However, a reduction in circulating levels of Lp(a) may also add to the beneficial effects of bariatric surgery on cardiovascular disease risk. The Lp(a) lowering effect of RYGB, observed in this study, is in accordance with results from previous studies. A recent meta-analysis including 13 studies and 1551 adults and adolescents revealed a significant decrease in circulating Lp(a) following different types of bariatric surgery (standardized mean difference; -0.438, 95% CI: -0.702, -0.174)(8). The heterogeneity between the studies was, however, large, and the mean (SD) Lp(a) levels at baseline ranged from 14.0 (3.65) to 258.2 (378) nmol/L. Meta-regression showed that there were no associations between

changes in Lp(a) levels **and** BMI change or **duration of follow-up** . Further, **in** 19

line with the present results, in a study involving 60 females, with and without overweight, a 9% increase in Lp(a) levels was shown after a 6-month period of calorie restriction(27). Also, in a cohort overlapping with the trial 2 cohort, Lp(a) increased in patients with overweight, with and without T2D, undergoing a calorie restricted diet for 3-4 months(7). However, other studies have showed no

change in Lp(a) levels after various dietary interventions aimed at weight loss 1

(28-30). Bariatric surgery and lifestyle-interventions did also differently influence FA levels. After bariatric surgery, plasma levels of total saturated FAs did not change form baseline, however plasma levels of palmitic acid decreased, while myristic acid, pentadecylic acid and stearic acid increased. The increased proportions of the saturated FAs myristic acid, pentadecylic acid and stearic acid following RYGB have previously been shown(31). Total plasma saturated FAs decreased after the lifestyle interventions, which was mainly due to a reduction in palmitic acid. This finding is in accordance with results from a previous 12-week randomized controlled trial comparing mild-calorie-restriction (minus 300 kcal/day) with a control diet(15). Calorie restricted diets typically include low levels of total fat and especially saturated fats, as was also the case for the participants in the lifestyle intervention groups in this study. However, dietary intake of saturated and also mono-unsaturated fats may not necessarily correlate with plasma levels as these dietary FAs are endogenously synthesized and remodeled(32). Plasma levels of the polyunsaturated FAs, on the other hand, correlate more strongly with dietary intake, and may better reflect dietary intake. Plasma levels of a number of n-3 FAs as well as the n-6 FA AA decreased after surgery, but increased during lifestyle interventions. Previous studies on the effect of calorie restriction and bariatric surgery on polyunsaturated FA levels showed somewhat conflicting results. In patients undergoing RYGB, the proportions of circulating n-3(33) and n-6 FAs(16) increased from baseline to 1 year after surgery. In contrast, among 13 women undergoing RYGB, phospholipid FA composition was similar to baseline levels at 6 months post-surgery, except for a decrease in content of EPA(17). Mild caloric restriction (minus 300 kcal/day) in 80 patients with overweight, resulted in greater reductions in plasma levels of some n-3 and n-6 FAs compared to control diet(15). Unfortunately, data on dietary intake was not collected, neither in

the lifestyle intervention groups nor in the bariatric surgery group. Thus, the strength of the relationship between changes in plasma FA levels and dietary intake cannot be assessed in the present study. However, one could speculate that the reduced intake of FAs in patients undergoing calorie restrictive diets would have a different effect on circulating FAs 17 and lipid metabolism compared with patients undergoing bariatric surgery where there is reduced absorption of FAs(34), changes in the composition of microbiota and often altered dietary preferences(35). The hypothesis of the present study was that bariatric surgery and lifestyle interventions would differently influence Lp(a) levels through a dissimilar effect on plasma FA levels. In the surgery-group, changes in Lp(a) were inversely associated with changes in saturated FAs and positively with changes in n-6 FAs, but there was no consistent pattern of associations between changes in Lp(a) and FA levels among the different lifestyle intervention groups.

An increased intake of total and saturated fats **has been** reported **to decrease Lp(a) levels** 1

(12, 36). However, mediation analysis failed to show an important role for any of the saturated FAs, in mediating the effect of surgery versus lifestyle intervention on Lp(a) levels in trial 1, an important result the current study adds to the existent knowledge. It has previously been shown that plasma levels of AA were positively associated with Lp(a) levels in patients suffering from familial hypercholesterolemia(9). AA is an antagonist of the

farnesoid X receptor (FXR)(37), **and FXR activation has been** found **to decrease Lp(a**) 3
levels(38). **Thus**

the increased levels of AA during the lifestyle interventions in the present study, could potentially have caused the observed increase in Lp(a) levels, whereas the decreased AA levels after surgery may have resulted in the observed reduced Lp(a) levels. However, according to the mediation analyses, the different effects of surgery and lifestyle interventions on Lp(a) levels were not explained by changes in plasma levels of AA, nor of any other FA or FA category. Bile acids also act as FXR agonists. Although bile acids were not measured in this study, previous studies have shown that circulating levels of bile acids are increased after RYGB(39, 40), which may partly explain the lowering of Lp(a) levels among the surgical patients. Interestingly, bile acid synthesis and levels have been shown to be increased in women with obesity, and to be normalized within 3 days on a caloric restriction diet(41). 18 Diet-induced lowering of bile acid production may therefore partly explain the increased Lp(a) levels observed during the lifestyle interventions. Future studies should determine whether the differential effects of surgery and lifestyle intervention on Lp(a) are mediated by changes in bile acid levels. Furthermore, one could speculate that exercise may have influenced the observed change in Lp(a) levels. A recent review reported that results from studies on the effect of exercise on Lp(a) levels have been inconsistent, with some reporting no effect while others have reported mildly increased or decreased levels(42). However, studies among younger individuals or patients with diabetes, showed more moderate Lp(a)-lowering effects by exercise. In trial 1, the participants underwent a physical activity intervention, and

the majority of the patients reported that they completed >3 hours of light physical activity per week and >3 hours of vigorous physical activity per week during follow- up 2

. Participants in the surgery group did not follow an exercise program prior to or following surgery. Exercise was also encouraged in trial 2, but the amount of physical activity performed did not change significantly from baseline. As Lp(a) increased during all lifestyle interventions even though only participants in the lifestyle-group of trail 1 followed an exercise program, it is less likely that the observed increase in Lp(a) was caused by exercise. Strenghts and limitations

The strengths of the present study are its prospective design and the use of two independent 1

trials with a relatively high number of patients with detailed analyses on both plasma FAs and Lp(a). Limitations include the non-randomized design, and plasma levels of Lp(a) and FAs being exploratory endpoints in both trials. Further, as

the participants had been referred to a tertiary care center, these findings may not be generalized to 1

all individuals with overweight and obesity. Of note, plasma Lp(a) levels were higher in trial 2 compared with trial 1, which may be partly explained by differences in analytical methods between the trials. Plasma samples from trial 2 were measured using a particle-enhanced immunoturbidimetric method 19 by DiaSys Diagnostic System, but also later re-analyzed in a sub-group of patients using Roche Diagnostics, as applied in trial 1. The median Lp(a) value in trial 2 was 13% higher using the DiaSys Diagnostic System versus using the Roche Diagnostics method. Repeated freezing/thawing cycles may influence Lp(a) levels in samples(43). However, the plasma samples were only frozen and thawed once before Lp(a) analyses in both trials, thus this is likely not an issue here. Another possible explanation may be differences in ethnicities between the trials. More than 55% of the participants in trial 2 were of non-White ethnicity, whereas 98% of the participants in trial 1 were White. Lp(a) levels are reported to vary across ethnicities, and people of non-White ethnicities are reported to have higher Lp(a) levels compared with those of White ethnicity(42). All participants in trial 2 had T2D compared with only 20% in trial 1. Previous studies have shown conflicting results regarding whether patients with T2D having higher or lower plasma Lp(a) levels than patients without T2D(7, 44, 45). Also, the participants in trial 2 were older than the participants in trial 1, and some studies suggest that Lp(a) increases with age, but the results are conflicting(46-50). Polyunsaturated FAs are also susceptible to degradation through freezing/thawing cycles. In trial 2, the samples were frozen only once prior to the FA analysis, while in trial, the samples were frozen twice before analysis. There is thus a possibility that there may have been some degradation of the polyunsaturated FAs in trial 1. Conclusion Lp(a) levels decreased in patients with obesity who underwent RYGB, but increased in patients with overweight or obesity undergoing lifestyle interventions. The Lp(a) lowering effect of bariatric

surgery has been shown in other studies, but the mechanisms behind this effect are unknown. The results of this study indicate that alterations of plasma levels of different FAs following RYGB do not explain any

changes in circulating **Lp(a) levels** . In addition, **changes in Lp(a) levels** 16

were not explained by changes in bodyweight. As 20 individuals with obesity have an increased risk of cardiovascular disease, the Lp(a) lowering effect of RYGB may be beneficial in this patient group. Also, one could speculate whether the reduction in circulating levels of Lp(a) partly explains the beneficial effects of bariatric surgery on cardiovascular disease. On the other hand, the increase in Lp(a) seen after lifestyle interventions may be potentially harmful to the individuals with obesity. If the mechanisms behind this were known, it becomes possible to take targeted actions to reduce this negative side effect. Our results make it more unlikely that manipulating fatty acid composition will contribute to the solution. Thus

future studies should clarify the mechanisms underlying the decrease **in Lp(a) levels** 1

after RYGB and the increase in Lp(a) levels following lifestyle interventions.

Long-term follow-up studies are also **required to determine whether elevated Lp(a) levels** , observed **after** 1

energy restricted diets,

are associated with an increased incidence **of cardiovascular disease in patients with** 13

overweight and obesity. 21 22 1 2 3 4 5 6 7 8 9 10 11 12 13 14 15 16 17 18 19 20 21 22 23 24 26 27 28 29 30 31 32 33 34 35 36 37 38 39 40 41 42 43 44 45 46 47 48 49 50 51 52 53 54 55 56 57 58 59 60 61 62 63 64 65 66 67 68 69 70 71 72 73 74 75 76 77 78 79 80 81 82 83 84 85 86 87 88 89 90 91 92 93 94 95 96 97 98 99 100 101 102 103 104 105 106 107 108 109 110 111 112 113 114 115 116 117 118 119 120 121 122 123 124 125 126 127 128 129 130 131 132 133 134 135 136 137 138 139 140 141 142 143 144 145 146 147 148 149 150 151 152 153 154 155 156 157 158 159 160 161 162 163 164 165 166 167 168 169 170 171 172 173 174 175 176 177 178 179 180 181 182 183 184 185 186 187 188 189 190 191 192 193 194 195 196 197 198 199 200 201 202 203 204 205 206 207 208 209 210 211 212 213 214 215 216 217 218 219 220 221 222 223 224 225 226 227 228 229 230 231 232 233 234 235 236 237 238 239 240 241 242 243 244 245 246 247 248 249 250 251 252 253 254 255 256 257 258 259 260 261 262 263 264 265 266 267 268 269 270 271 272 273 274 275 276 277 278 279 280 281 282 283 284 285 286 287 288 289 290 291 292 293 294 295 296 297 298 299 300 301 302 303 304 305 306 307 308 309 310 311 312 313 314 315 316 317 318 319 320 321 322

323 324 325 326 327 328 329 330 331 332 333 334 335 336 337 338 339 340 341 342 343 344 345 346 347 348 349 350 351 352 353 354 355 356 357 358 359 360 361 362 363 364 365 366 367 368 369 370 371 372 373 374 375 376 377 378 379 380 381 382 383 384 385 386 387 388 389 390 391 392 393 394 395 396 397 398 399 400 401 402 403 1 2 3 4 5 6 9 11 12 14 15 16

sources:

1 146 words / 3% - Internet
Berk, K.A.C. (Kirsten). "Weight Loss and Weight Loss Maintenance in Type 2 Diabetes", urn:ISBN:978-94-92683-73-1, 2017

2 46 words / 1% - Internet
Gjevestad, Espen. "Comparative effects of lifestyle intervention, low calorie diet and bariatric surgery on weight loss and arterial stiffness. Non-randomized clinical trials including treatment seeking morbidly obese patients"

3 28 words / < 1% match - Internet
Narverud, I. (Ingunn), Bogsrud, M.P. (Martin P.) et al. "Lipoprotein (a) concentration is associated with plasma arachidonic acid in subjects with familial hypercholesterolemia", 'Cambridge University Press (CUP)', 2019

4 20 words / < 1% match - Internet
Yahya, R. (Reyhana). "Dyslipidemia beyond LDL", Many features of dyslipidemia are missed by the standard lipid panel. I investigated the use of advanced lipoprotein profiling in the diagnosis of dyslipidemia. I found that normoglycemic first degree relatives of type 2 diabetes (T2D) patients have lower high density lipoprotein (HDL) levels than controls from non T2D families. I also found that the drug lomitapide decreases all atherogenic lipoproteins, but does not affect HDL function. Lipoprotein (a) (Lp(a)) is a risk factor for the development of cardiovascular disease (CVD), but there are no therapies available yet to solely reduce Lp(a) levels. I investigated the effect of widely used therapies, 2019

5 26 words / < 1% match - Internet from 21-Oct-2022 12:00AM
translational-medicine.biomedcentral.com

6 23 words / < 1% match - Internet from 14-May-2019 12:00AM
www.acrwebsite.org

7 20 words / < 1% match - Internet from 24-Aug-2022 12:00AM
bmcmusculoskeletdisord.biomedcentral.com

8 14 words / < 1% match - Internet from 12-Sep-2021 12:00AM
digibug.ugr.es

9 13 words / < 1% match - Internet from 20-Sep-2020 12:00AM
onlinelibrary.wiley.com

10 12 words / < 1% match - Crossref
D. Hofso. "Obesity-related cardiovascular risk factors after weight loss: a clinical trial comparing gastric bypass surgery and intensive lifestyle intervention", European Journal of Endocrinology,

11/01/2010

11 12 words / < 1% match - Internet from 03-Oct-2022 12:00AM
foodandnutritionresearch.net

12 12 words / < 1% match - Internet from 05-Oct-2022 12:00AM
vital.seals.ac.za

13 11 words / < 1% match - Internet from 26-Feb-2022 12:00AM
pesquisa.bvsalud.org

14 11 words / < 1% match - Internet from 03-Nov-2022 12:00AM
www.alliedacademies.org

15 10 words / < 1% match - Crossref
"Poster Presentations", Obesity Reviews, 07/09/2010

16 10 words / < 1% match - Crossref
Akihiro Nakamura, Masanori Kanazawa, Yuta Kagaya, Masateru Kondo, Kenjiro Sato, Hideaki Endo, Eiji Nozaki. "Plasma kinetics of mature PCSK9, furin-cleaved PCSK9, and Lp(a) with or without administration of PCSK9 inhibitors in acute myocardial infarction", Journal of Cardiology, 2020

17 10 words / < 1% match - Crossref
Scanu, A.M.. "Postprandial lipoprotein (a) response to a single meal containing either saturated or @w-3 polyunsaturated fatty acids in subjects with hypoalphalipoproteinemia", Metabolism, 199212

18 10 words / < 1% match - Internet from 20-Sep-2022 12:00AM
pure.rug.nl

19 10 words / < 1% match - Internet from 19-Nov-2022 12:00AM
www.researchgate.net
